# Universal resilience patterns in labor markets

**DOI:** 10.1038/s41467-021-22086-3

**Published:** 2021-03-30

**Authors:** Esteban Moro, Morgan R. Frank, Alex Pentland, Alex Rutherford, Manuel Cebrian, Iyad Rahwan

**Affiliations:** 1 Departamento de Matemáticas & GISC, Universidad Carlos III de Madrid, Leganés, Spain; 2 Media Laboratory, Massachusetts Institute of Technology, Cambridge, MA USA; 3 Institute for Data, Systems, and Society, Massachusetts Institute of Technology, Cambridge, MA USA; 4 Sociotechnical Systems Research Center, Massachusetts Institute of Technology, Cambridge, MA USA; 5 Department of Informatics and Networked Systems, School of Computing and Information, University of Pittsburgh, Pittsburgh, PA USA; 6 Digital Economy Lab, Institute for Human-Centered AI, Stanford University, Stanford, CA USA; 7 Center for Humans & Machines, Max Planck Institute for Human Development, Berlin, Germany

**Keywords:** Environmental economics, Applied mathematics, Interdisciplinary studies

## Abstract

Cities are the innovation centers of the US economy, but technological disruptions can exclude workers and inhibit a middle class. Therefore, urban policy must promote the jobs and skills that increase worker pay, create employment, and foster economic resilience. In this paper, we model labor market resilience with an ecologically-inspired job network constructed from the similarity of occupations’ skill requirements. This framework reveals that the economic resilience of cities is universally and uniquely determined by the connectivity within a city’s job network. US cities with greater job connectivity experienced lower unemployment during the Great Recession. Further, cities that increase their job connectivity see increasing wage bills, and workers of embedded occupations enjoy higher wages than their peers elsewhere. Finally, we show how job connectivity may clarify the augmenting and deleterious impact of automation in US cities. Policies that promote labor connectivity may grow labor markets and promote economic resilience.

## Abstract (web summary)

Recent technological, social, and educational changes are profoundly impacting our work, but what makes labour markets resilient to those labour shocks? Here, the authors show that labour markets resemble ecological systems whose resilience depends critically on the network of skill similarities between different jobs.

## Introduction

Like ecosystems^1,2^, the cities with adaptable labor markets are best prepared for the future of work. So what makes cities adaptable? This question remains open because the typical study of urban labor at equilibrium obfuscates responses to out-of-equilibrium disruptions. For example, most automation research relies on occupation-level estimates of technological exposure^3,4^. But, labor markets are heterogeneous systems in which skills^5^, jobs, geographies^6,7^, and sectors all interact^8^. Accordingly, models built on empirical skills data may better capture worker mobility and better identify the sources of urban adaptability through the interdependencies of workplace skills^9,10^. Analogous strategies predict resilience to shocks in ecological systems (e.g., changing acidity or temperature levels) from the density of mutualistic interdependencies between species—independent of population dynamics at equilibrium^11,12^. Similar network models have been applied in a variety of economic systems including global socioeconomic systems^13^, regional economies^14^, banking systems^15^, and interfirm worker mobility^16^ through an analogy connecting ecological mutualism to economic complementarity. In these studies, the interdependencies between cities, banks, and firms undergird systemic resilience to shocks even though dynamics in those systems are very different. Despite their success in other economic domains, studies of interdependencies between skills and occupations remain absent from urban labor studies on economic resilience.

How strong is the analogy between labor market resilience and the resilience of ecosystems? To study this question, we first expand existing labor theory using an analogy between the overlapping skill requirements of occupations within a labor market and the mutualistic interactions between species within an ecosystem. Since labor markets and ecosystems may be governed by different dynamics, how can ecological methods apply to labor market resilience? Recent studies demonstrate the prominent role of structure in determining ecological resilience independent of the ecosystem’s population-level dynamics^11,12^. Thus, we ignore the different equilibrium dynamics that govern a labor market or an ecosystem, and we apply analogous measures to occupation networks in US cities constructed from employment distributions and skills data provided by the US Department of Labor. While existing foundational studies have identified similar structures in urban labor markets^17–20^, we are not aware of any study empirically relating these economic structures to economic resilience following a labor shock. Thus, we expand this body of work by applying our theoretical framework to measure labor market resilience compared to the unemployment experienced across US cities following the Great Recession. Finally, having empirically validated our measure following an actual labor disruption, we apply our framework to understand the impact of another labor disruption: automation via computerization.

## Results

Traditional labor market models (e.g., job matching theory^21^) lack the granularity required by this ecological view of labor. In general, the job matching function *M*(*U*, *V*) describes the dynamics of employment, *E*, as a function of unemployed workers, *U*, filling job vacancies, *V*, according to

1 $$\frac{dE}{dt}=-\lambda U+M(U,V),\quad \frac{dU}{dt}=\lambda E-M(U,V)$$

where *λ* represents the rate of job match dissolution. Employment is increased as job seekers fill job vacancies through the matching function^22^ between unemployed workers and available jobs; most research models this process as *M*(*U*, *V*) ∝ *U*^*γ*^*V*^1−*γ*^ where *γ* ∈ [0.5, 0.7]. However, the abstract nature of this model obfuscates the frictions that limit workers’ career mobility^23^. In particular, skill mismatch^21^ is difficult to identify without refining the model with empirical skill interdependencies.

To describe career mobility between occupations, the model must be extended to treat each occupation *j* as its own submarket^9,24,25^ with unemployed workers, *U*_j_, and job vacancies, *V*_j_. This requires a revised matching function *M*(*U*_i_, *V*_j_) that respects frictions in the flow of workers between occupations *i* and *j*. To this end, recent studies using the O*NET database from the US Bureau of Labor Statistics (BLS) have revealed skill polarization^26^, the impact of automation in cities^3^, and demonstrated how similar skill requirements are predictive of worker transitions between occupations^27,28^.

**Fig. 1:**
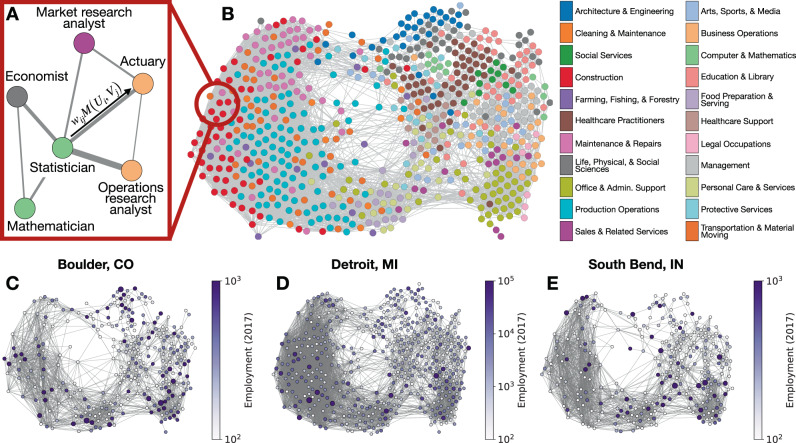
The job connectivity for visualizing urban labor markets. **A** A schematic for skill similarity *w*_ij_ and the job matching process. **B** The job network constructed from skill similarity scores. Each occupation is represented by a circle colored according to sector. This job network visualization is filtered to links with *w*_ij_ > 0.70; the complete job network was used in all analysis. **C**–**E** Example cities are projected onto subsets of the job network based on employment by occupation in 2017.

Here, we use O*NET skills data to calculate the pairwise similarity of skill requirements for occupations *i* and *j* according to *w*_ij_ = 1 − ∣∣**O**_*i*_ − **O**_*j*_∣∣_2_ where **O**_*j*_ denotes the skill vector of occupation *j* (see Methods to learn more about this skills database from the BLS). We normalize skill similarity scores so that *w*_ij_ ∈ [0, 1]. Our results throughout are robust to alternative skill similarity calculations (see Supplementary Note 2), varying *γ* (see Supplementary Note 5), and even small perturbations in the set of skills used to calculate the similarity (see Supplementary Note 3). We model skill similarity scores as a job network where occupation pairs are connected with weighted links according to *w*_ij_. Consistent with previous work^26^, the resulting network reveals a polarized aggregate structure (see Fig. 1B) suggesting that the topology of skill interdependencies relates to job polarization. In this new formulation, job matching happens according to $M({U}_{{\mathrm{i}}},{V}_{{\mathrm{j}}})\propto {w}_{{\mathrm{ij}}}{U}_{{\mathrm{i}}}^{\gamma }{V}_{{\mathrm{j}}}^{1-\gamma }$. Since *E*_j_ ∝ *V*_j_ across occupations (see Supplementary Note 1), we use eq. (1) to obtain

2 $$\frac{d{E}_{{\mathrm{j}}}}{dt} =-\lambda {E}_{{\mathrm{j}}}+\alpha \mathop{\sum}\limits _{i\in {\rm{Jobs}}}{w}_{{\mathrm{ij}}}{E}_{{\mathrm{j}}}^{\gamma }{U}_{{\mathrm{i}}}^{1-\gamma }\\ \frac{d{U}_{{\mathrm{j}}}}{dt} =\lambda {E}_{j}-\alpha \mathop{\sum} \limits_{i\in {\rm{Jobs}}}{w}_{{\mathrm{ij}}}{E}_{{\mathrm{i}}}^{\gamma }{U}_{{\mathrm{j}}}^{1-\gamma }.$$

Note that this model conserves the total number of workers (i.e., ∑_j∈Jobs_ *E*_j_ + *U*_j_ is a constant). We assume that a worker’s skills are approximated by the skills required by their most recent employment (i.e., ${{\bf{O}}}_{{E}_{{\mathrm{j}}}}={{\bf{O}}}_{{U}_{{\mathrm{j}}}}$) and we assume that the sets of occupations and skills are fixed. Under these assumptions, the model is best suited for describing responses to shocks rather than long-term forces that shape labor markets, such as education, retraining programs, and innovation.

Equation  (2) resembles models of ecological mutualism^29–31^. As with mutualistic ecosystems, labor markets in cities that employ workers in occupations with overlapping skill requirements create positive spillover effects that can bolster the resilience of the labor market. For example, if employment decreases for some occupation, then other similar occupations could support displaced workers without costly retraining. Therefore, as in ecological modeling^11,12^, the density of connections between occupations within a city could indicate greater economic resilience to shocks, including unemployment shocks, the automation of workplace tasks, or other major disruptions. Essentially, city’s with more connections between occupations will be more resilient to labor shocks.

We test this hypothesis by applying eq. (2) to employment data for each US city. For city *c*, we seed the model with occupational employment according to the BLS to determine ${E}_{{\mathrm{j}}}^{{\mathrm{c}}}$ and assume that *α* and *λ*, which represent national labor trends, are constant across cities and occupations. If ${E}_{{\mathrm{j}}}^{{\mathrm{c}}}=0$, then we assume city *c* is unable to support workers in occupation *j* and we set ${w}_{{\mathrm{ij}}}^{{\mathrm{c}}}=0$ for each *i* ∈ Jobs; otherwise, we take ${w}_{{\mathrm{ij}}}^{{\mathrm{c}}}={w}_{{\mathrm{ij}}}$. Our results are robust to alternative definitions of the minimum number of jobs to set ${w}_{{\mathrm{ij}}}^{{\mathrm{c}}}=0$, see Supplementary Note 3 for more details. Thus, each city has its own job network constructed as a subset of the complete job network (see Fig. 1B for the complete job network and Fig. 1C–E for examples of job networks in cities). These job networks are extremely high-dimensional representations of urban labor markets that may seem intractable at first. For example, a city with *N* unique occupations has $({{N}\atop{2}})$ skill similarity scores (i.e., links in that city’s job network). However, despite job network size and complexity, those multidimensional systems can be simplified to an effective one-dimensional model based on average nearest-neighbor activity^11^. Letting ${w}_{{\mathrm{j}}}^{{\mathrm{c}}}={\sum }_{{\mathrm{i}}\in {\rm{Jobs}}}{w}_{{\mathrm{ij}}}^{{\mathrm{c}}}$ (called occupation *j*’s embeddedness) and ${W}^{{\mathrm{c}}}={\sum }_{{\mathrm{i,j}}\in {{\rm{Jobs}}}^{2}}{w}_{{\mathrm{ij}}}^{{\mathrm{c}}}$, we define the effective variables^11^

3 $${E}_{{\rm{eff}}}^{{\mathrm{c}}}=\left(\mathop{\sum} _{{\mathrm{j}}\in {\rm{Jobs}}}{E}_{{\mathrm{j}}}^{{\mathrm{c}}}{w}_{{\mathrm{j}}}^{{\mathrm{c}}}\right)/{W}^{{\mathrm{c}}},\quad\! {U}_{{\rm{eff}}}^{{\mathrm{c}}}=\left(\mathop{\sum} _{{\mathrm{j}}\in {\rm{Jobs}}}{U}_{{\mathrm{j}}}^{{\mathrm{c}}}{w}_{{\mathrm{j}}}^{{\mathrm{c}}}\right)/{W}^{{\mathrm{c}}},\quad \,\text{and}\,\quad\! {w}_{{\rm{eff}}}^{{\mathrm{c}}}=\left(\mathop{\sum} _{{\mathrm{j}}\in {\rm{Jobs}}}{({w}_{{\mathrm{j}}}^{{\mathrm{c}}})}^{2}\right)/{W}^{{\mathrm{c}}}$$

and reduce eq.  (2) to

4 $$\frac{d{E}_{{\rm{eff}}}^{c}}{dt}	=-\lambda {E}_{{\rm{eff}}}^{c}+\alpha {w}_{{\rm{eff}}}^{c}{({E}_{{\rm{eff}}}^{c})}^{\gamma }{({U}_{{\rm{eff}}}^{c})}^{1-\gamma }\\ \frac{d{U}_{{\rm{eff}}}^{c}}{dt}	=\lambda {E}_{{\rm{eff}}}^{c}-\alpha {w}_{{\rm{eff}}}^{c}{({E}_{{\rm{eff}}}^{c})}^{\gamma }{({U}_{{\rm{eff}}}^{c})}^{1-\gamma }.$$

These effective variables capture the expected long-term dynamics in each city given our model and rely heavily on each city’s job network. In particular, ${w}_{{\rm{eff}}}^{c}$ captures the job network connectivity between occupations and uniquely determines simulated employment levels. Using the effective system, each city has two potential long-term outcomes: systemic collapse with ${E}_{{\rm{eff}}}^{c}=0$ or a healthy system with the fraction of employed workers given by

5 $${\hat{E}}_{{\rm{eff}}}^{c}=\frac{1}{1+\left(\right.\lambda /(\alpha {w}_{{\rm{eff}}}^{c}){\left)\right.}^{1/(1-\gamma )}}.$$

See Methods section for the complete derivation.

**Fig. 2:**
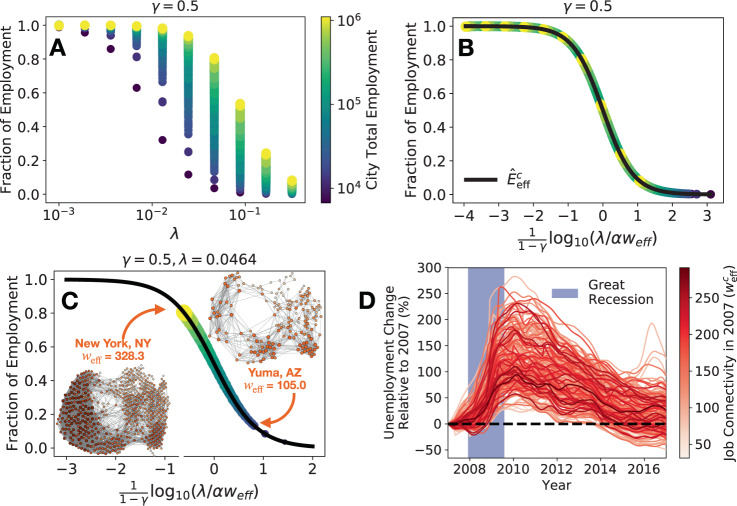
Job connectivity determines a universal trend in urban resilience and predicts unemployment during the Great Recession. **A** The steady-state solutions of the simulation model for each city while varying the rate of job match dissolution *λ*. **B** Similar to (**A**) but controlling for the job connectivity in each city ${w}_{{\rm{eff}}}^{c}$. Solid line is the analytically-derived employment rate at equilibrium. **C** The equilibrium solutions of our model for each city for *λ* = 0.046 and job network projections for two example cities given by occupations with nonzero employment in each city. In (**A**–**C**), symbol size and color represent total employment in the city. The color bar provided in (**A**) also applies to (**B**) and (**C**). **D** Unemployment by US city during and after the Great Recession. Lines are colored by the city’s job connectivity in 2007.

The effective model yields several important insights into the economic resilience of urban labor markets. First, although the model allows for system collapse, this outcome is unattainable if ${E}_{{\rm{eff}}}^{c}\,> \, 0$, which is the fortunate case for each city we modeled. Second, despite varying in size and geography, cities’ expected employment (i.e., eq. (5)) follows universal dynamics determined by ${w}_{{\rm{eff}}}^{c}$. For each rate of job matching dissolution (*λ*), the variation in simulated long-term employment levels (see Fig. 2A) collapses to a single line after controlling for ${w}_{{\rm{eff}}}^{c}$ (see Fig. 2B. See Supplementary Note 7 for simulation details.).

These results suggest that job connectivity is critical to a city’s economic resilience. Cities with greater ${w}_{{\rm{eff}}}^{c}$ have a larger fraction of employed workers ${\hat{E}}_{{\rm{eff}}}^{c}$ after simulation (see Fig. 2B). Fixing *λ* clarifies this relationship and shows how cities can have different stationary solutions under the same exogenous forces (see Fig. 2C). For example, under a large country-wide dissolution rate of jobs (*λ* = 0.046), some cities, such as New York, NY, are able to resist changes to net employment, but other cities, such as Yuma, AZ, experience significant decreases in their fraction of employed workers according to simulations. The results in Fig. 2 are robust to variations in *λ* (see Supplementary Note 4) and alternative choices of *γ* (see Supplementary Note 5).

Beyond simulation, is there an empirical relationship between job connectivity and urban responses to employment shocks? To externally validate our model, we compare city’s job connectivity in 2007 to their peak unemployment rate during the Great Recession (December 2007 to June 2009) using Local Area Employment Statistics (see Fig. 2D). In general, larger cities experienced lower unemployment rates (see Table 1, Model 1), but city’s total employment in 2007 was not very predictive. This result suggests that the thickness of their labor markets^32^ do not determine their response to the employment shock. Rather, various aspects of labor diversity better explain urban responses. For instance, city’s with greater job connectivity also experienced lower unemployment rates (see Table 1, Model 2) following a stronger relationship than city size alone. We expand on this result to include other aspects of labor diversity such as employment diversity (i.e., the Shannon entropy of employment by occupation, *H*^c^) and occupational diversity (i.e., the number of unique occupations, *N*^c^). Employment diversity is given by ${H}^{c}=-{\sum }_{j\in {\rm{Jobs}}}{p}_{j}^{c}\cdot {\mathrm{log}\,}_{10}({p}_{j}^{c})$ where ${p}_{j}^{c}$ is occupation *j*’s fraction of employment in city *c*. Even after controlling for employment diversity and occupational diversity, job connectivity significantly improves predictions of peak unemployment rates during the Great Recession (see Table 1, Model 3 compared to Model 4 & 5). Combined, this evidence highlights the critical role of job connectivity in city’s economic resilience to labor shocks, like the Great Recession.

**Table 1:** Linear regression models predicting the peak unemployment rate in US cities during the Great Recession. Using 2007 employment data from the year before the recession, labor diversity measured through urban employment distributions (*H*^c^), occupation diversity (*N*^c^), and job connectivity (*w*_eff_) predict labor responses to Great Recession. Variables were centered and standardized prior to analysis.

Dependent variable: Peak unemployment Rate during the Great Recession					
Variable	Model 1	Model 2	Model 3	Model 4	Model 5
${\mathrm{log}\,}_{10}$ Total Employment (*T*^*c*^)	−0.175^*^		0.514	0.521	0.510
Job connectivity (${w}_{{\rm{eff}}}^{c}$)		−0.218^**^		−3.952^***^	−4.372^***^
Occupation diversity (*N*^c^)			0.210	4.005^***^	4.559^***^
Employment diversity (*H*^c^)			−0.988^***^	−0.833^***^	−0.847^***^
${w}_{{\rm{eff}}}^{c}\times {T}^{c}$					−0.344
${w}_{{\rm{eff}}}^{c}\times {N}^{c}$					0.039
${w}_{{\rm{eff}}}^{c}\times {H}^{c}$					0.267^*^
*R*^2^	0.031	0.047	0.190	0.234	0.262
adjusted *R*^2^	0.026	0.043	0.178	0.218	0.235

*p*_value_ < 0. 1^*^, *p*_value_ < 0.01^**^, *p*_value_ < 0.001^***^

**Fig. 3:**
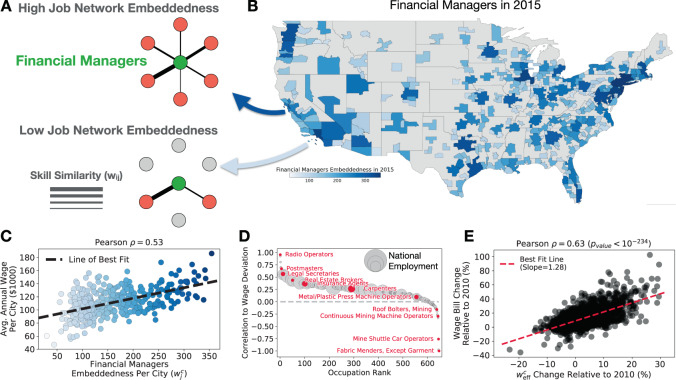
Workers earn higher wages when their occupation is embedded in their local labor market which increases job connectivity and the city’s wage bill. **A** A schematic explaining occupation embeddedness ($${w}_{{\mathrm{j}}}^{{\mathrm{c}}}$$) in a city’s job network projection. **B** US cities colored according to the embeddedness of Financial Managers in 2015. **C** In cities where the occupation is more embedded, Financial Managers earn higher wages than their peers in other cities. Point color corresponds to Financial Manager’s embeddedness in each city (i.e., corresponds to the city’s color in **B**)). **D** For 75% of workers nationwide, workers of the same occupation earn higher wages in cities where the occupation has greater embeddedness. See Supplementary Note 8 for a controlled regression analysis and embeddedness maps for other occupations. **E** Using 2010 as a baseline, cities that increased job connectivity ($${w}_{{\rm{eff}}}^{c}$$) saw corresponding increases in wage bill ($). There is a data point for each city and each year from 2011 to 2017. See Supplementary Note 9 for regression analysis and a similar analysis of year-to-year dynamics.

Since job connectivity may promote economic resilience to labor disruptions, how can policy makers and individuals leverage connectivity and job network projections? One way is to compare the embeddedness (${w}_{j}^{c}$) of a single occupation in the job network projection of each city (e.g., see Fig. 3A, B). For 75% of US employment and 96% of US occupations, workers of a given occupation that is more embedded in their city’s labor market earn higher annual wages than workers of the same occupation in other cities (see Fig. 3C, D). We observe evidence for this embeddedness wage premium even after additionally controlling for a occupation’s employment share, educational requirements, city fixed effects, and occupation fixed effects (see Supplementary Note 8 for more details). This suggests workers may benefit from employment opportunities in cities where their employment would boost the city’s job connectivity. In general, cities that increased their job connectivity from 2010 through 2017 saw an increase in their wage bill (i.e., total wages. See Fig. 3E). Thus, policy makers may be able to grow their local labor market through targeted investment in the companies and industries that employ workers of embedded occupations which increases the overall job connectivity of the city.

**Fig. 4:**
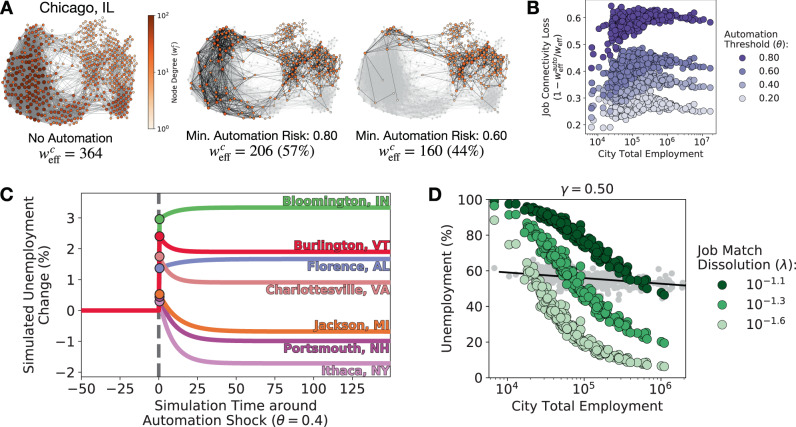
Automation decreases job connectivity in cities and yields diverse employment responses. **A** The job network projection of Chicago, IL in 2014 (${w}_{{\rm{eff}}}^{c}=307$). Job connectivity (${w}_{{\rm{eff}}}^{c}$) decreases as occupations are removed from the labor market according to each occupation’s automation risk^4^. **B** The change in job connectivity across cities of different sizes with varying automation exposure. ${w}_{{\rm{off}}}^{auto}$ denotes the city’s job connectivity after removing occupations from automation. **C** Simulated city responses to an unemployment shock from automation. If occupation *i* has probability of automation greater than *θ* = 0.4, then all workers of *i* immediately become unemployed and we set ${w}_{ij}^{c}=0$ for each other occupation *j*. Each simulation begins at steady-state using the city’s complete job network projection in 2014. Each color represents a different US city. **D** The simulated fraction of unemployed workers in each US city compared to total employment. Gray points correspond to expected job impact from automation in each US city^3^ (solid line is a linear fit). Colored points correspond to the steady-state solution of simulations while varying the rate of job match dissolution (*λ*).

Besides the Great Recession, can job connectivity tell us about other labor shocks, such as technology and automation? To explore, we combine our model with estimates of occupation-level exposure to computerization^4^ and simulate urban employment (see Supplementary Note 7 for simulation details). An occupation *i* is automated if its probability of computerization is above some minimum threshold *θ*. All workers of an automated occupation are immediately unemployed and job seekers can no longer become employed in that occupation (i.e., *w*_ij_ = 0 for each *i* ≠ *j*). With Chicago, IL as an example (see Fig. 4A), decreasing *θ* corresponds to greater automation and decreasing job connectivity in cities (see Fig. 4B). Not only are jobs lost because of automation, but job networks become sparser and less resilient.

In Fig. 4C, we simulate eq.  (2) for each using 2014 employment data until the simulation reaches a steady-state, then we automate each occupation whose probability of computerization exceeds *θ* = 0.4. This produces a sudden increase unemployment in each city. However, as we continue the simulation of urban responses after the automation shock, we observe a rich set of responses including the possibility of decreased unemployment. For example, Burlington, VT, and Bloomington, IN, experience similar changes in unemployment immediately after the automation shock, but Burlington is able to recover after the shock while Bloomington experiences increased unemployment. Similarly, Charlottesville, VA, experiences greater initial automation shock than Florence, AL, but is able to recover in the long-term while Florence experiences worsening unemployment. Interestingly, several cities experience increased unemployment immediately after the automation shock, but actually experience lower unemployment in the long-term compared to their unemployment prior to the shock. The reason for this variety of behaviors lies in how job connectivity is affected by automation in different cities. Job connectivity in cities like Charlottesville or Burlington is high even after some jobs are automated, while it drops significantly in places likes Florence or Bloomington. As a result, the latter labor markets cannot accommodate the initial automation shock.

In general, these simulations offer a new tool to policy makers for distinguishing between potentially substitutive technological impact and augmentation. First, consistent with previous studies of automation and cities^3^, small cities tend to face greater impact (i.e., greater initial disruption) from automation. The gray points in Fig. 4D demonstrate the exposure to automation in each US city using methods from^3^. However, simulating long-term employment dynamics using eq.  (2) after the initial shock suggests higher-order dynamics. Cities can recover, or worsen, after the initial impact. Although our simulation of automation shocks is simplistic (e.g., rarely are whole occupations automated^8,33,34^), this type of insight sheds light on the growing economic and labor disparity between large US cities and smaller rural areas^3,35^ and offers a potential pathway to forecast labor dynamics and resilience given the uncertain nature of future technologies.

## Discussion

As complex interdependent systems, urban labor markets are more than the sum of individual occupations or sectors. Specifically, this study maps the dependencies between occupations in urban labor markets based on occupational skill requirements and demonstrates how the topology of these connections between occupations relates economic resilience to job network connectivity and relates workers’ wages to occupational embeddedness. Job connectivity and occupational embeddedness are topological features that rely entirely on the job network in each city, and insights based on these features would not be calculable in the absence of the job network (e.g., using employment for each industry or job title in isolation). Further, our analysis of job connectivity and occupation embeddedness is careful to control for potential confounders from traditional network-independent variables that might limit the salience of our conclusions (see Supplementary Notes 8 and 9 for details). These refinements to traditional job matching theory provide insight into economic resilience to labor disruptions, including technological change and the Great Recession. In particular, our results suggest policy that promotes the occupational embeddedness of workers may increase job network connectivity, as well as the economic resilience and size of their local labor market (e.g., as measured by total wage bill). Future research could develop more sophisticated spatial econometric models^36^ and identify specific policy instruments for promoting job connectivity and occupational embeddedness, and quantify the most impactful use of these policy options.

## Methods

### BLS

Number of jobs and wage estimates by sector and city were obtained from the US BLS for the 2017 year^37^. Cities are defined as the core-based statistical areas (CBSA) the New England cities and town areas (NECTA) included in the US Census. By definition the CBSAS or NECTA are geographical areas that consist of one of more counties (or equivalents) anchored by an urban center of at least 10,000 people plus adjacent counties that are socioeconomical tied to the urban center. Some of the cities/areas span over different states, like the ones in New York or Boston, for example. The BLS data contains information about the number of workers and wage estimates of 772 occupations and 422 cities. Occupations are described by the Standard Occupational Classification 6 digits^38^.

### The O*NET database

The O*NET Database is a data product of the US BLS^39^. The O*NET program is the “nation’s primary source of occupational information,” and is continually updated through surveys of various workers from each occupation in the Standard Occupation Classification (SOC) taxonomy. The O*NET database identifies the distinguishing characteristics of each occupation.

Every occupation requires a different mix of knowledge, skills, and abilities, and is performed using a variety of activities and tasks. In this study, we focus on the abilities, interests, knowledge, skills, work activities, work contexts, education, training, and experience O*NET variables to identify the importance of 232 different workplace skills for 775 different occupations. These O*NET variables result from a composition of surveys each conducted with varying Likert scales; thus, we normalize the responses from each survey so that each O*NET variable *s* has some real-valued importance to occupation *j* denoted by *O*(*j*, *s*) ∈ [0, 1] such that *O*(*j*, *s*) = 1 indicates that *s* is essential to *j* and *O*(*j*, *s*) = 0 indicates that *s* is irrelevant to *j*. Letting *S* denote the set of 232 O*NET variables, we also use **O**_*j*_ = […, *O*(*j*, *s*), … ]_*s*∈*S*_ to represent the skill vector associated with occupation *j*.

### Unemployment data

Unemployment rates were obtained from the US BLS Local Area Unemployment Statistics^40^. Specifically we used the seasonally adjusted metropolitan area estimates by month.

### Deriving effective long-term employment

Equation (4) describes a system with two fixed points that represent the potential long-term employment dynamics of a given city’s labor market. The first occurs when *E*_eff_ = 0 which indicates a systemic collapse where employment in that city has disappeared. However, the instability of this fixed point suggests it is difficult for real-world labor systems to realize this abysmal future. Rather, the second—and more preferable—fixed point describes the long-term fraction of workers with employment (denoted ${\hat{E}}_{{\rm{eff}}}^{c}$) given a city *c*’s job connectivity ${w}_{{\rm{eff}}}^{c}$.

To derive this second fixed point associated with ${\hat{E}}_{{\rm{eff}}}$ at *E*_eff_ and *U*_eff_, we begin with

6 $$0={\dot{E}}_{{\rm{eff}}}=-\lambda {E}_{{\rm{eff}}}+\alpha {w}_{{\rm{eff}}}{({E}_{{\rm{eff}}}^{* })}^{\gamma }{({U}_{{\rm{eff}}}^{* })}^{1-\gamma }$$

which we rewrite as

7 $${U}_{{\rm{eff}}}^{* }={E}_{{\rm{eff}}}^{* }{\left(\frac{\lambda }{\alpha {w}_{{\rm{eff}}}}\right)}^{1/(1-\gamma )}.$$

Recalling that the model conserves the total number of workers, we have

8 $${E}_{{\rm{eff}}}^{* }(0)+{U}_{{\rm{eff}}}^{* }(0)={E}_{{\rm{eff}}}^{* }(t)+{U}_{{\rm{eff}}}^{* }(t)$$

9 $$=\left(1+{\left(\frac{\lambda }{\alpha {w}_{{\rm{eff}}}}\right)}^{1/(1-\gamma )}\right){E}_{{\rm{eff}}}^{* }(t)$$

which can be rewritten as

10 $${E}_{{\rm{eff}}}^{* }=\frac{{E}_{{\rm{eff}}}^{* }(0)+{U}_{{\rm{eff}}}^{* }(0)}{1+{\left(\frac{\lambda }{\alpha {w}_{{\rm{eff}}}}\right)}^{1/(1-\gamma )}},$$

which implies the fraction of employed workers is given by

11 $${\hat{E}}_{{\rm{eff}}}=1/\left(1+{\left(\frac{\lambda }{\alpha {w}_{{\rm{eff}}}}\right)}^{1/(1-\gamma )}\right).$$

## Supplementary information

Supplementary Information

## Data Availability

Data supporting the findings of this study are available within the paper and its supplementary information files. All employment and skills data used in this study are publicly available from the US BLS. City-level estimates of automation impact are available in the supplementary materials of^3^.

## Code availability

Standard numerical integration methods were used to integrate the equations of model (2). Standard linear regression methods are used to fit the data. The python codes used to analyze data and run the simulations of model (2) are available from the authors upon reasonable request.
