# Supplementary material for: Universal resilience patterns in labor markets
Source: Nat Commun. 2021 Mar 30;12:1972. doi: 10.1038/s41467-021-22086-3 (PMC8009945; doi:10.1038/s41467-021-22086-3)
Supplement: Supplementary file 1 — Supplementary Information [file 41467_2021_22086_MOESM1_ESM.pdf]

Supplementary Information: Universal resilience patterns in labor markets

Esteban Moro[1,2,3,4,†,*], Morgan R. Frank[2,4,5,6,†], Alex Pentland[2,3,4], Alex Rutherford[7], Manuel Cebrian[7], & Iyad Rahwan[7,*]

[1] *Departamento de Matemáticas & GISC, Universidad Carlos III de Madrid, 28911 Leganés, Spain*

[2] *Media Laboratory, Massachusetts Institute of Technology, Cambridge, MA 02139, USA*

[3] *Institute for Data, Systems, and Society, Massachusetts Institute of Technology, Cambridge, MA 02139, USA*

[4] *Sociotechnical Systems Research Center, Massachusetts Institute of Technology, Cambridge, MA 02139, USA*

[5] *Department of Informatics and Networked Systems, School of Computing and Information, University of Pittsburgh, Pittsburgh, PA, USA*

[6] *Digital Economy Lab, Institute for Human-Centered AI, Stanford University, Stanford, CA, USA*

[7] *Center for Humans & Machines, Max Planck Institute for Human Development, Lentzeallee 94, 14195, Berlin, Germany*

† *These authors contributed equally.*

**To whom correspondence should be addressed; E-mail:* emoro@math.uc3m.es *or* rahwan@mpib-berlin.mpg.de

Supplementary Notes

Supplementary Note 1: **Job Vacancies are Proportional to Employment** **3**

Supplementary Note 2: **The Universality of Skill Complexity with Alternative Job Network Construction** **4**

Supplementary Note 3: **Robustness of the job network connectivity definition** **6**

Supplementary Note 4: **Sensitivity to Job Match Dissolution λ** **10**

Supplementary Note 5: **Sensitivity to Varying γ** **11**

Supplementary Note 6: **Examples of City Projections onto the Job Network** **15**

Supplementary Note 7: **Simulating Labor Flows within City** **17**

Supplementary Note 8: **Job Network Embeddedness and Worker Wages** **19**

Supplementary Note 9: **Job Connectivity and Wage Bill Growth** **30**

Supplementary References **34**

Supplementary Note 1: Job Vacancies are Proportional to Employment

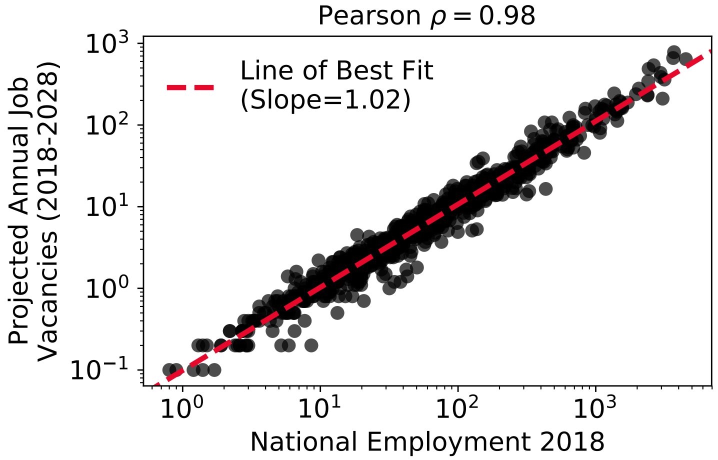

Figure S1: Projections of annual job vacancies by occupation for 2018 to 2028 are proportional to national employment according to employment projections from the US Bureau of Labor Statistics.

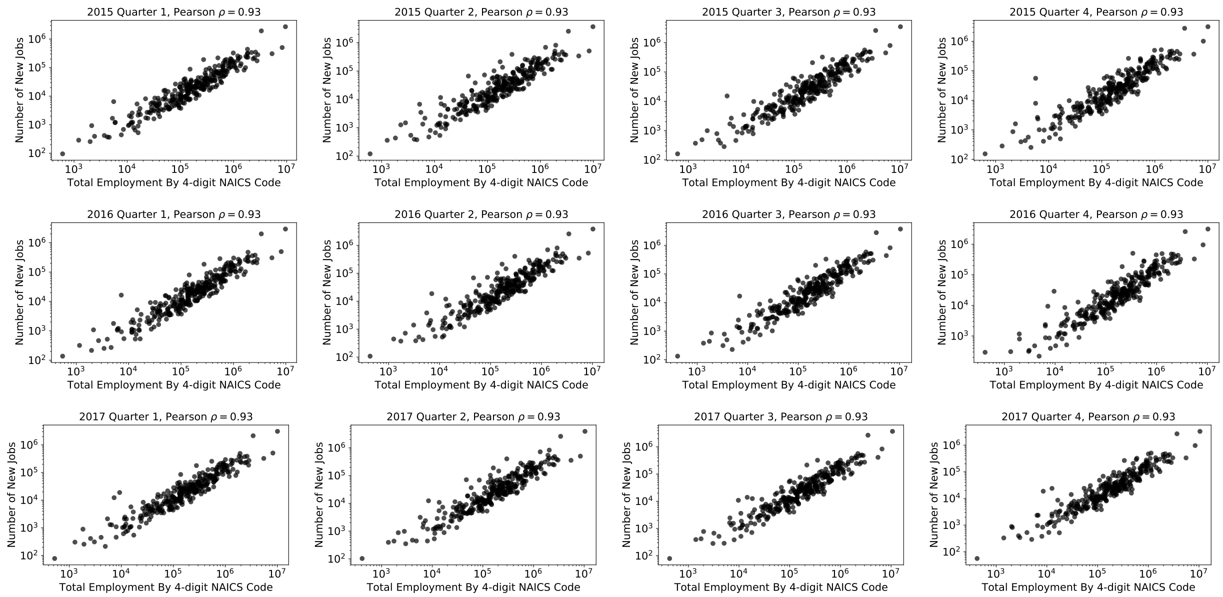

Figure S2: For each quarter in 2015, 2016, and 2017, the number of new jobs (i.e., internal or external hiring) by 4-digit NAICS Industry code was proportional to the industry's total employment. This analysis used Longitudinal Employer-Household Dynamics data from the US Census Bureau.

Supplementary Note 2: The Universality of Skill Complexity with Alternative Job Network Construction

In this section, we consider the job network constructed with an alternative skill similarity metric. Specifically, we measure the Jaccard similarity of the O*NET skills required by each occupation according to

$$\text{jaccard}(i,j) = \frac{\sum\limits_{s \in Skills} \min(O(i,s), O(j,s))}{\sum\limits_{s \in Skills} \max(O(i,s), O(j,s))},$$

where $O(i,s)$ is the relative weight of skill s in job i. The universality of the skill complexity of cities is consistent for this alternative job network construction (see Fig. S3 and Fig. S4).

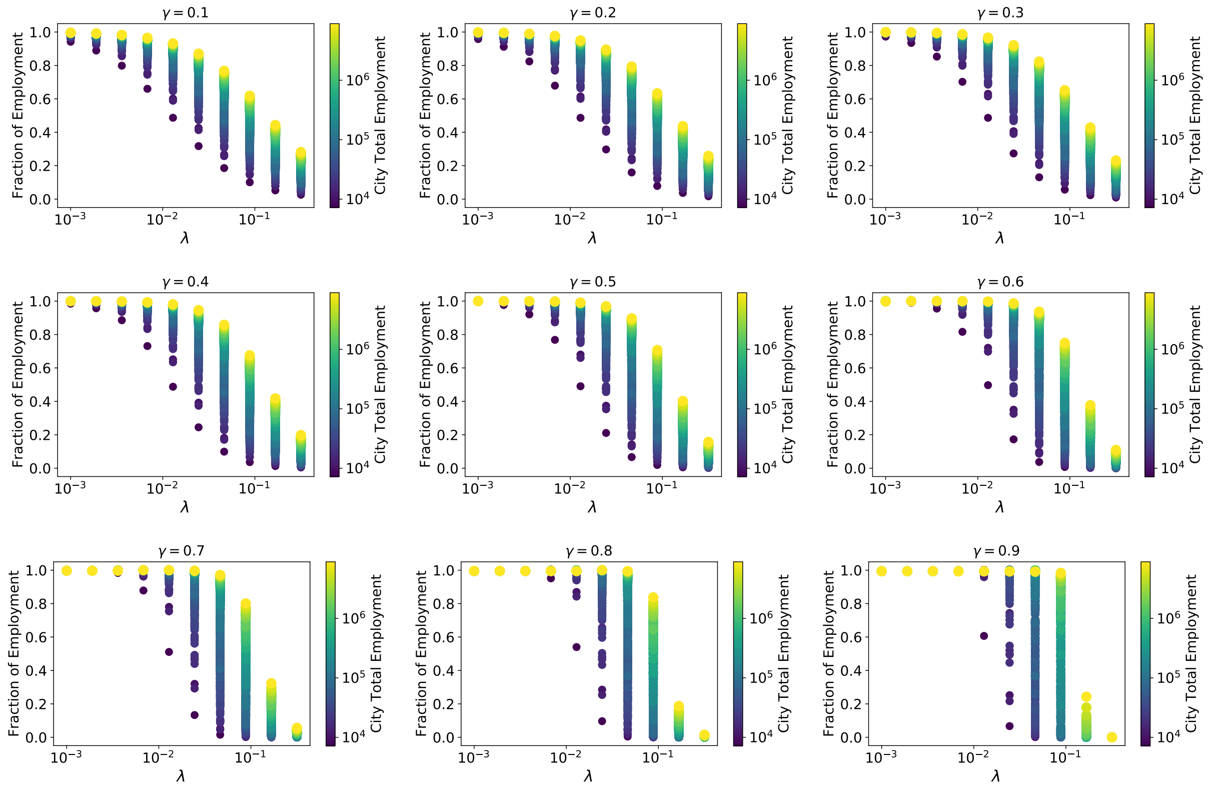

Figure S3: The equilibrium solutions of our model for each city while varying γ and the rate of job match dissolution λ. Each panel represents a different choice of γ. Symbol size and color represent total employment in the city. As an alternative method, we consider the job-job network constructed from Jaccard skill similarity.

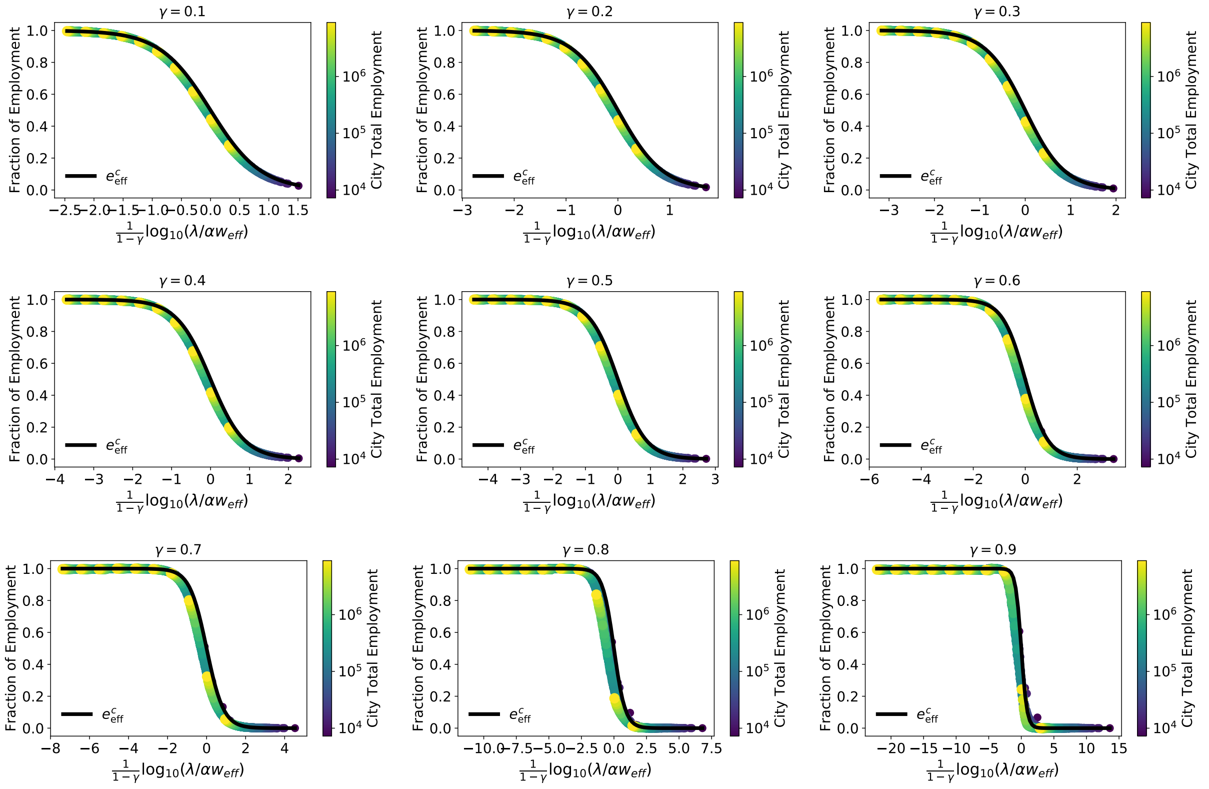

Figure S4: The equilibrium solutions of our model for each city while varying γ and the rate of job match dissolution λ after controlling for the skill matching complexity in each city w^c_{eff}. Each panel represents a difference choice of γ. As an alternative method, we consider the job-job network constructed from Jaccard skill similarity. Symbol size and color represent total employment in the city. Solid line is the analytically-derived equilibrium solution e^c_{eff}.

Supplementary Note 3: Robustness of the job network connectivity definition

Our *job network connectivity* w_{eff} depends on the definition of the job network w_{ij}. Here we study the robustness of w_{eff} with respect to the different assumptions made to build the job network.

Firstly we made $w_{ij}^c = 0$ in city c for jobs i with the number of jobs $E_j^c = 0$ in the BLS data. Since BLS does not report occupations i that have less than 30 people employed by city we are effectively using a threshold in our definition, i.e. $w_{ij}^c = 0$ if $E_j^c < \theta$. Figure S5 shows that w_{eff} does not change when that threshold is increased beyond 30. In fact, cross-correlation of the values are pretty high (around 90%) for different values of the threshold.

On the other hand, the construction of the job network depends on the specific composition of skills by jobs given in the O*NET tables. The question is whether the actual value of w_{eff} for a given city depends critically on that specific composition which could affect it dramatically if information about skills is missing or incomplete. Or simply if skills are redefined for a different market or country. To test this we have recalculated the values of w_{eff} for the different cities in our dataset by removing randomly a set of skills in the definition of jobs. Figure S6 shows that the values of w_{eff} are pretty robust against the actual set of skills used to calculate the job network.

In summary, both tests performed in this section show that our definition of w_{eff} and, in turn, our results do not depend critically on the assumptions made to construct the job network.

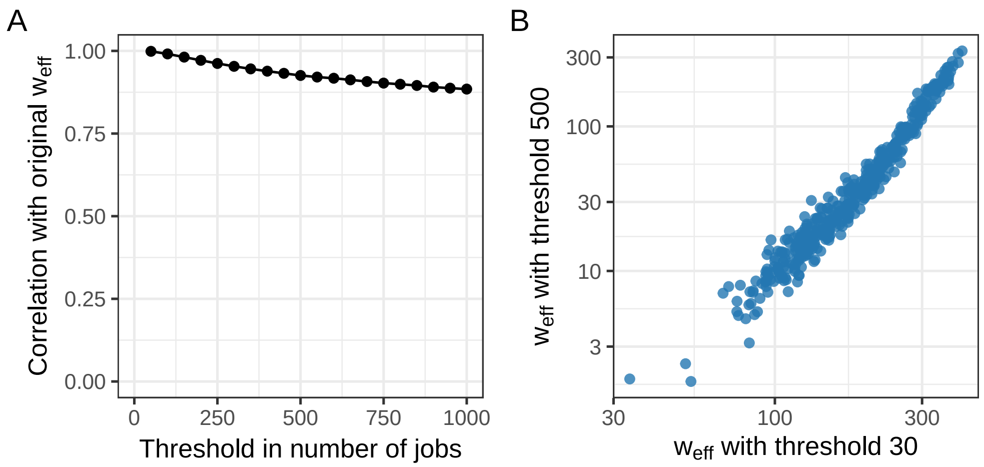

Figure S5: A) Correlation of between the original w_{eff} (for different cities) and the one calculated using a different minimum threshold in the number of jobs. B) Comparison between the w_{eff} values of each city using 30 (as in the BLS data) and 500 as the minimum threshold in the number or jobs.

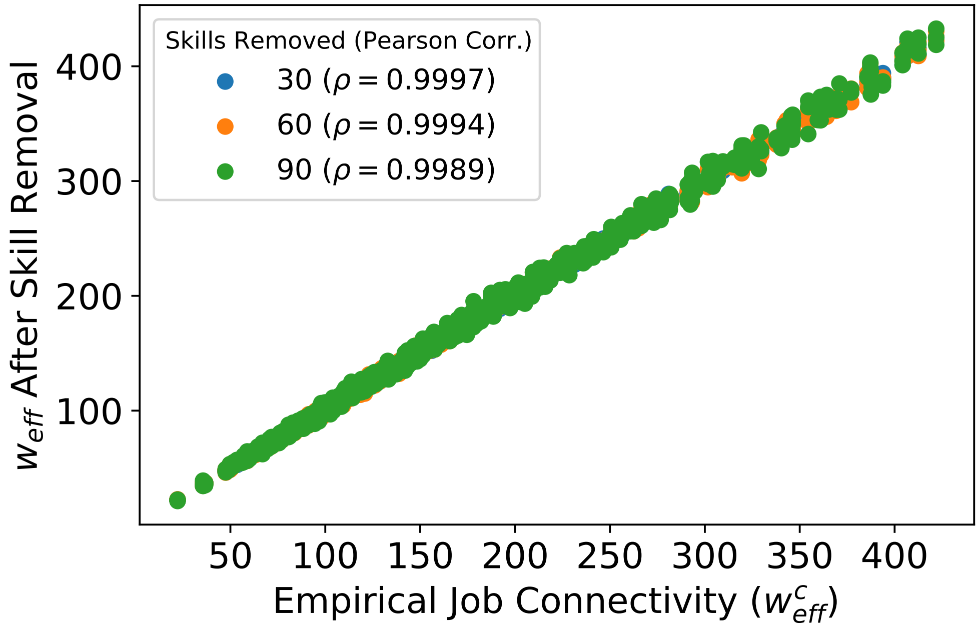

Figure S6: Comparison between the original w_{eff} (including all skills) and the one calculated after randomly removing a number of skills in the definition of jobs.

Supplementary Note 4: Sensitivity to Job Match Dissolution λ

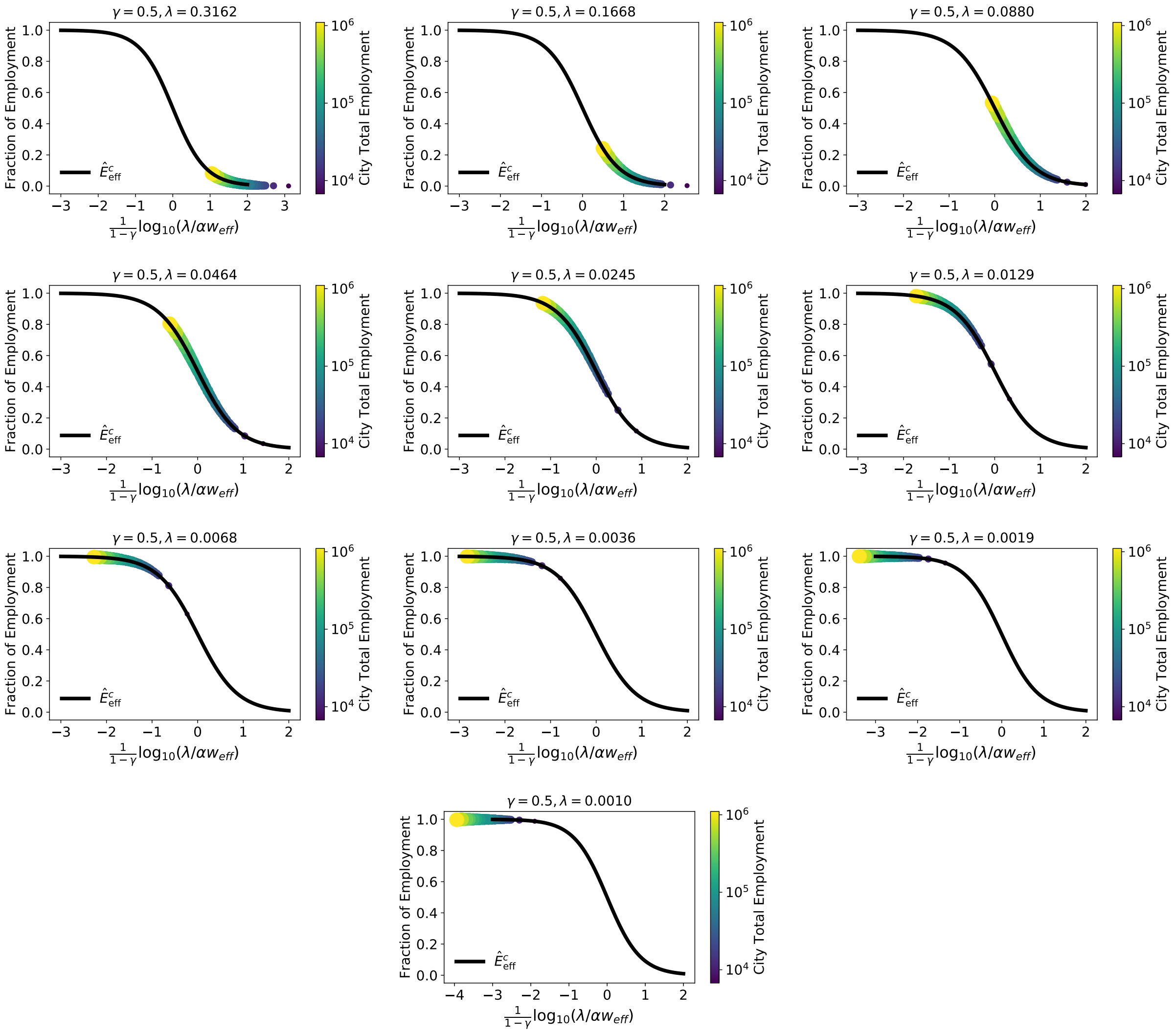

Figure S7: The equilibrium solutions of our model for each city for $\gamma = 0.50$ while varying λ and controlling for the skill matching complexity in each city w^c_{eff}. Each panel represent a different choice of λ. Symbol size and color represent total employment in the city. Solid line is the analytically-derived equilibrium solution e^c_{eff}.

Supplementary Note 5: Sensitivity to Varying γ

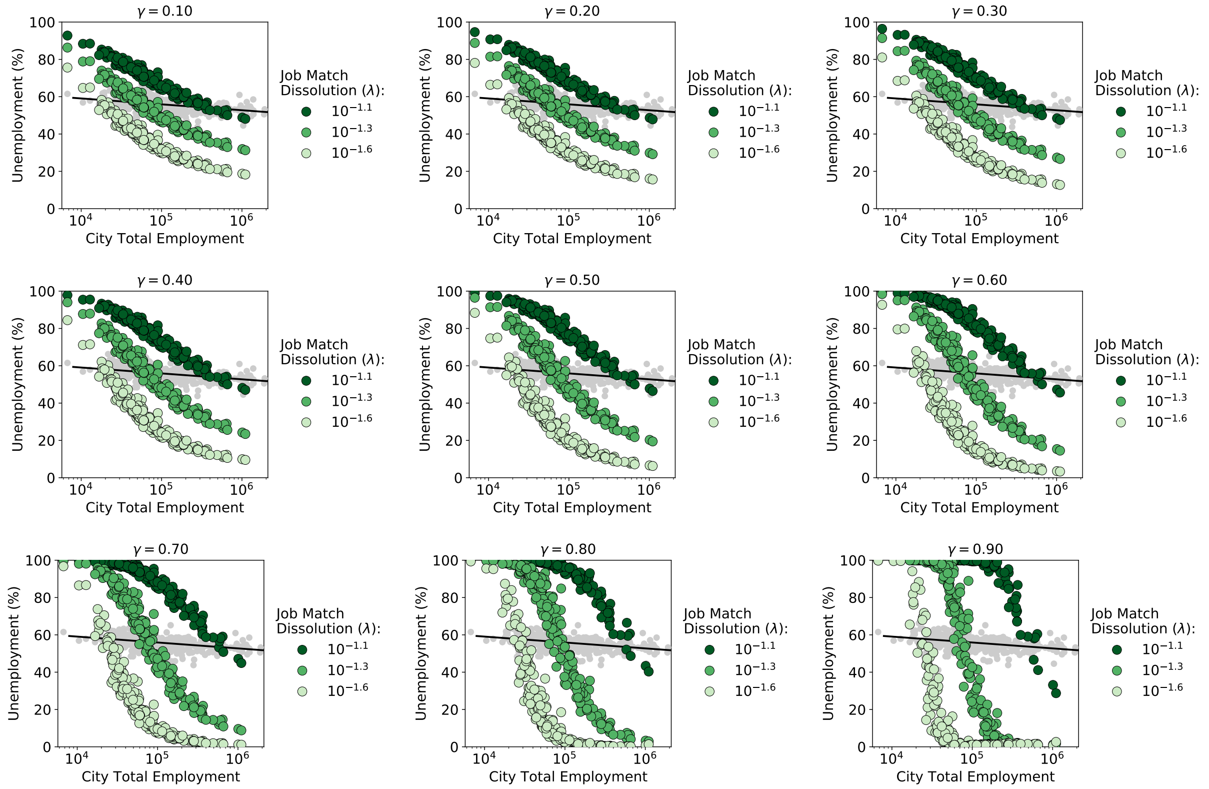

Figure S8: Simulated unemployment rates across city sizes while varying γ. Each panel represents a different choice of γ. Color represents the rate job match dissolution λ. Grey dots represent the expected job impact from automation in each city using estimates from [1]; the solid line indicates the line of best fit.

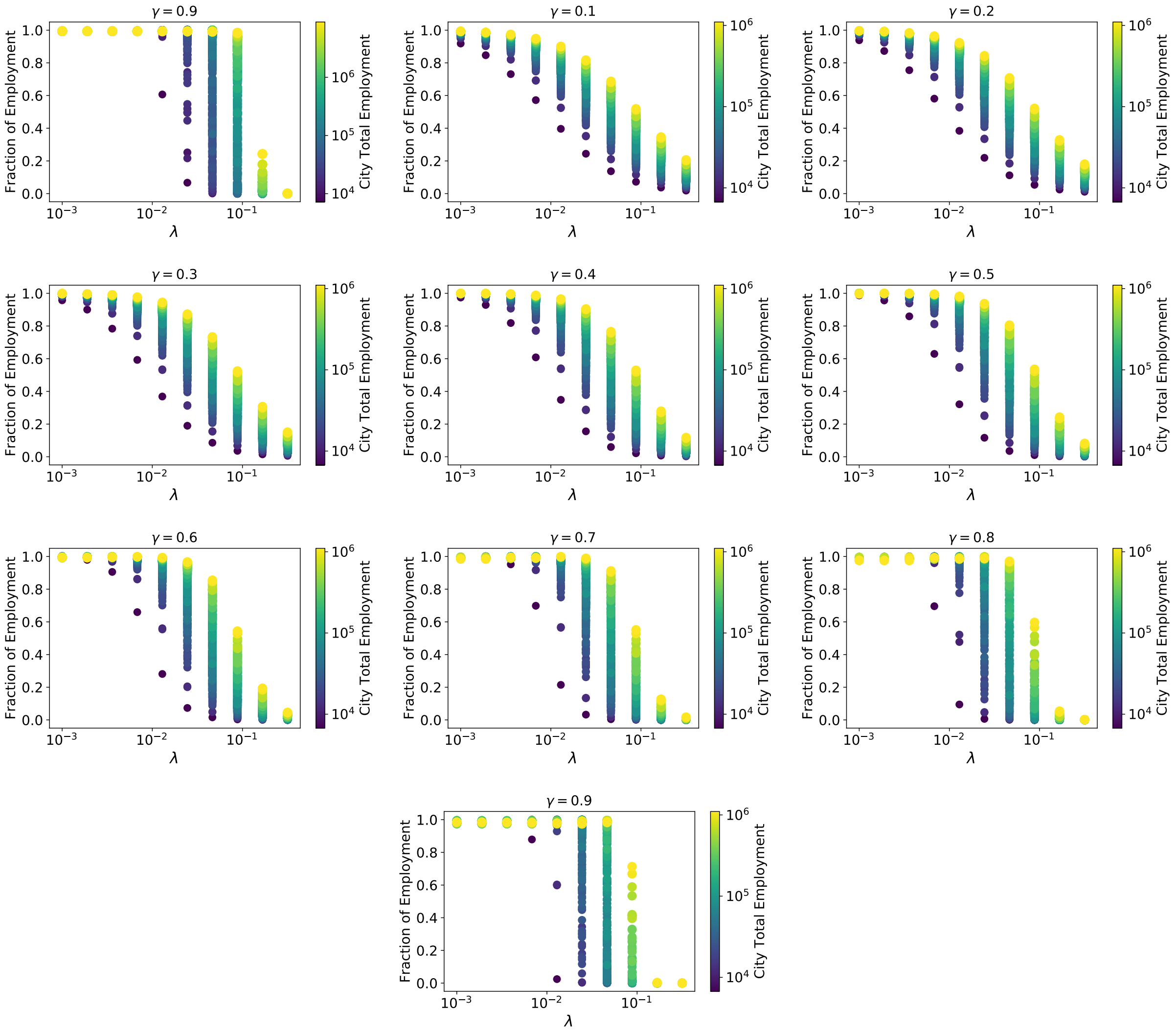

Figure S9: The equilibrium solutions of our model for each city while varying γ and the rate of job match dissolution λ. Each panel represents a different choice of γ. Symbol size and color represent total employment in the city.

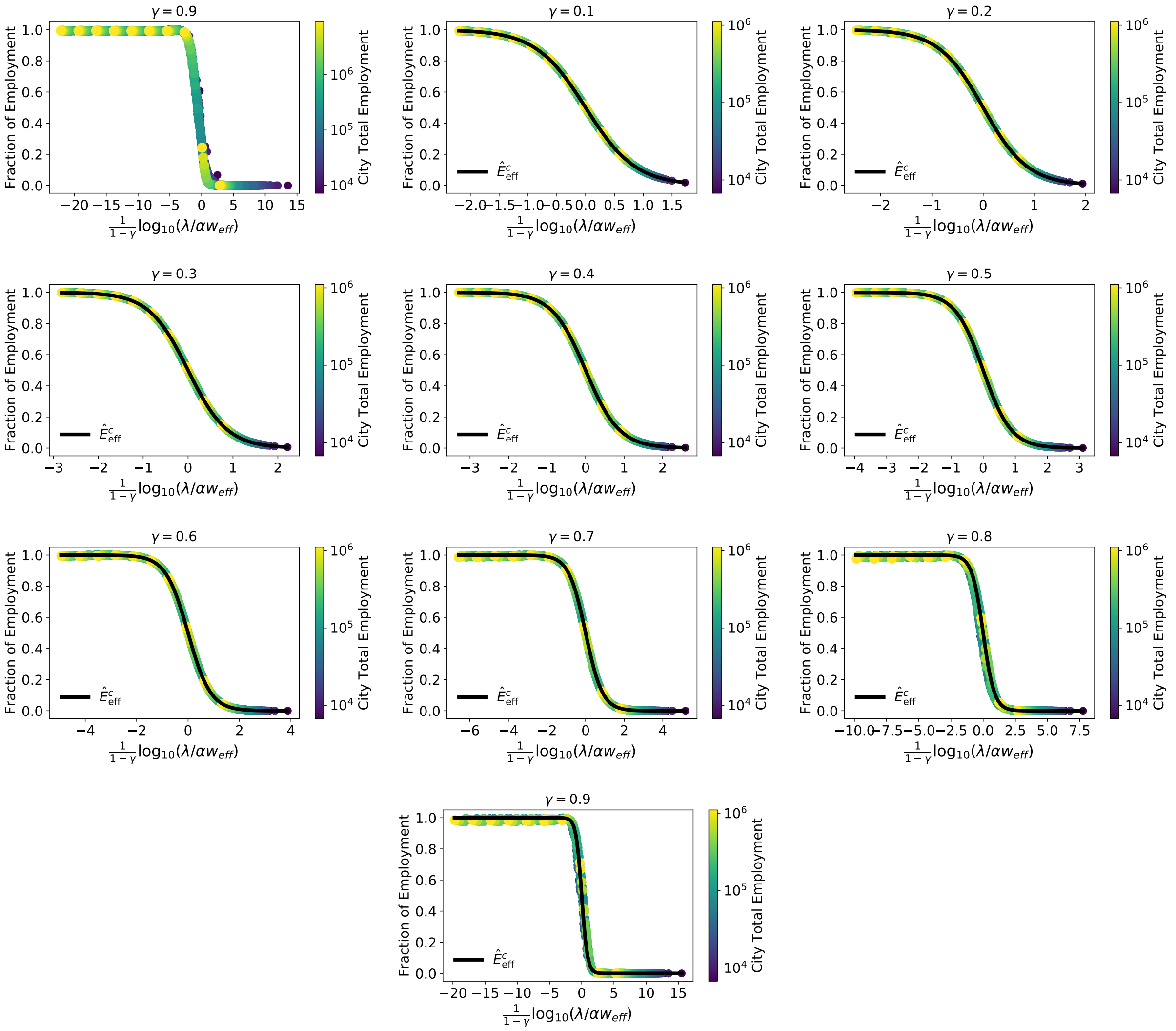

Figure S10: The equilibrium solutions of our model for each city while varying γ and the rate of job match dissolution λ after controlling for the skill matching complexity in each city w^c_{eff}. Each panel represents a different choice of γ. Symbol size and color represent total employment in the city. Solid line is the analytically-derived equilibrium solution e^c_{eff}.

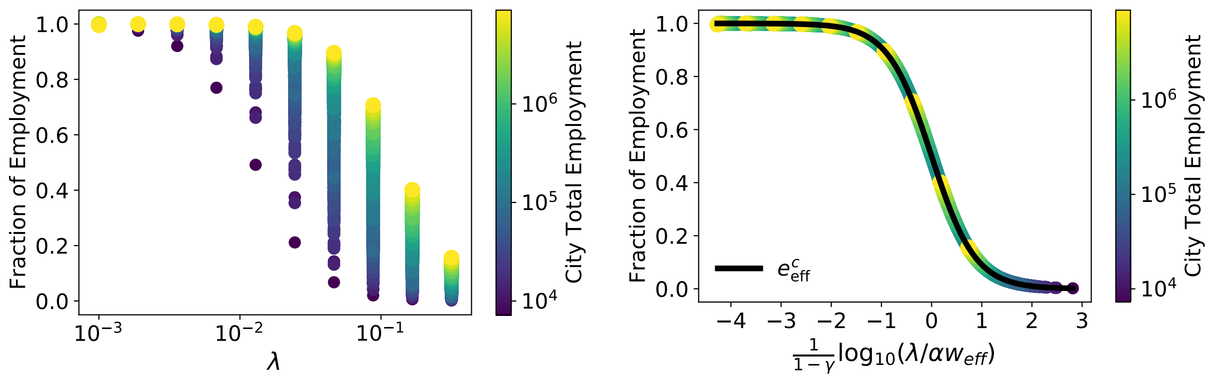

Figure S11: The universality of skill complexity persists when γ varies. After 10 independent trials where $\gamma \in [0.4, 0.6]$ is selected uniformly at random, we plot the average results to compare to the results from the main text where $\gamma = 0.5$ is fixed.

Supplementary Note 6: Examples of City Projections onto the Job Network

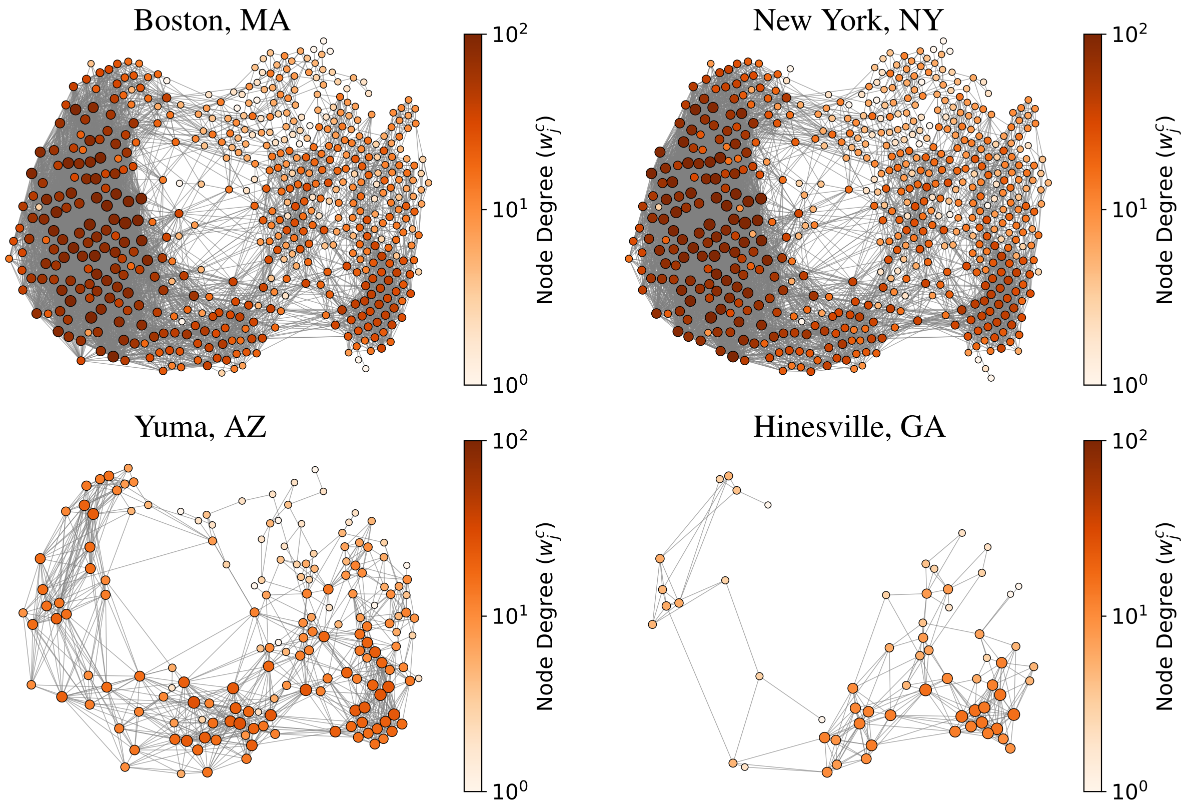

Figure S12: Examples of cities projected onto the job network. City projections are the sub-networks defined by the occupations with non-zero employment in a city. Each occupation is represented by a circle colored according to its weighted degree w_j^c.

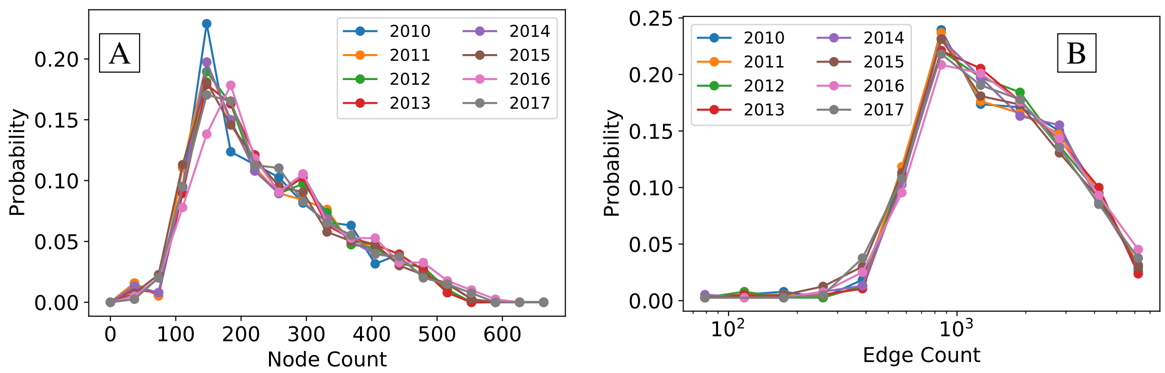

Figure S13: **(A)** Distribution of node counts for city's job network projections by year. **(B)** Distribution of edge counts for city's job network projections by year.

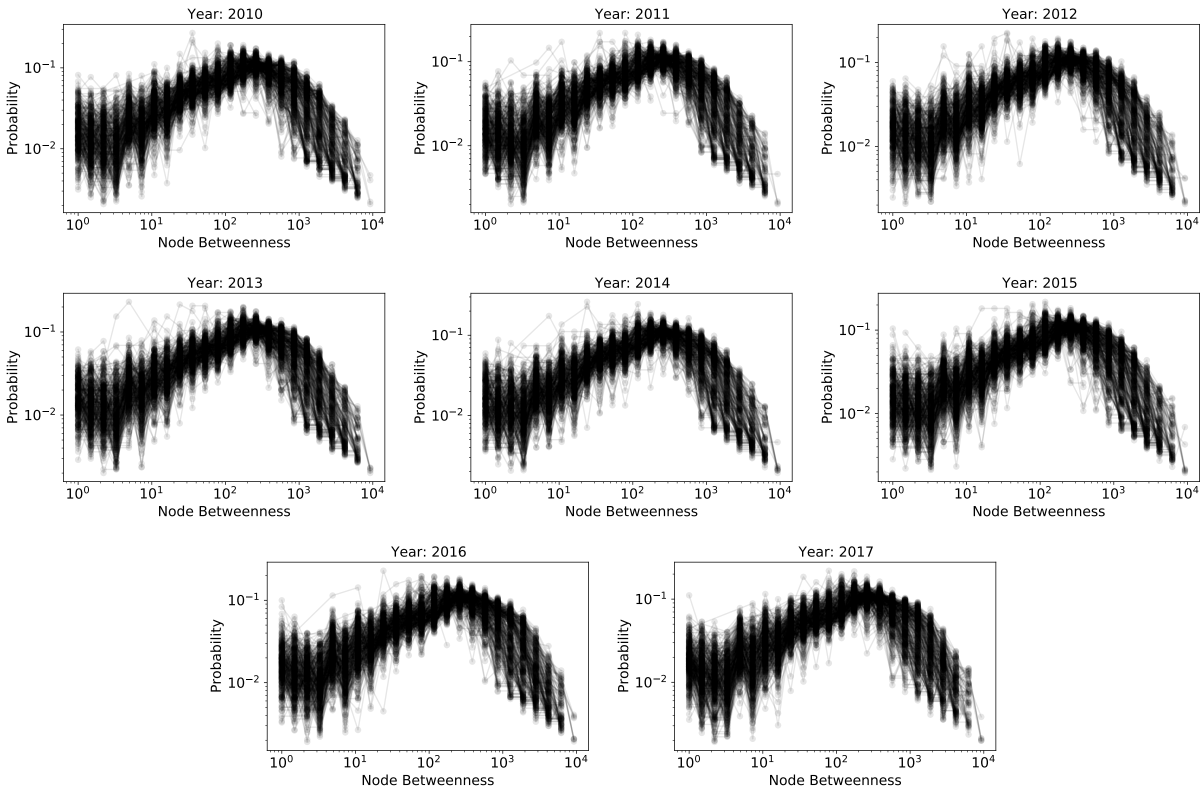

Figure S14: The distribution of node betweenness score for each city's job network projection calculated in a few example years. For each year, one line is plotted for each city.

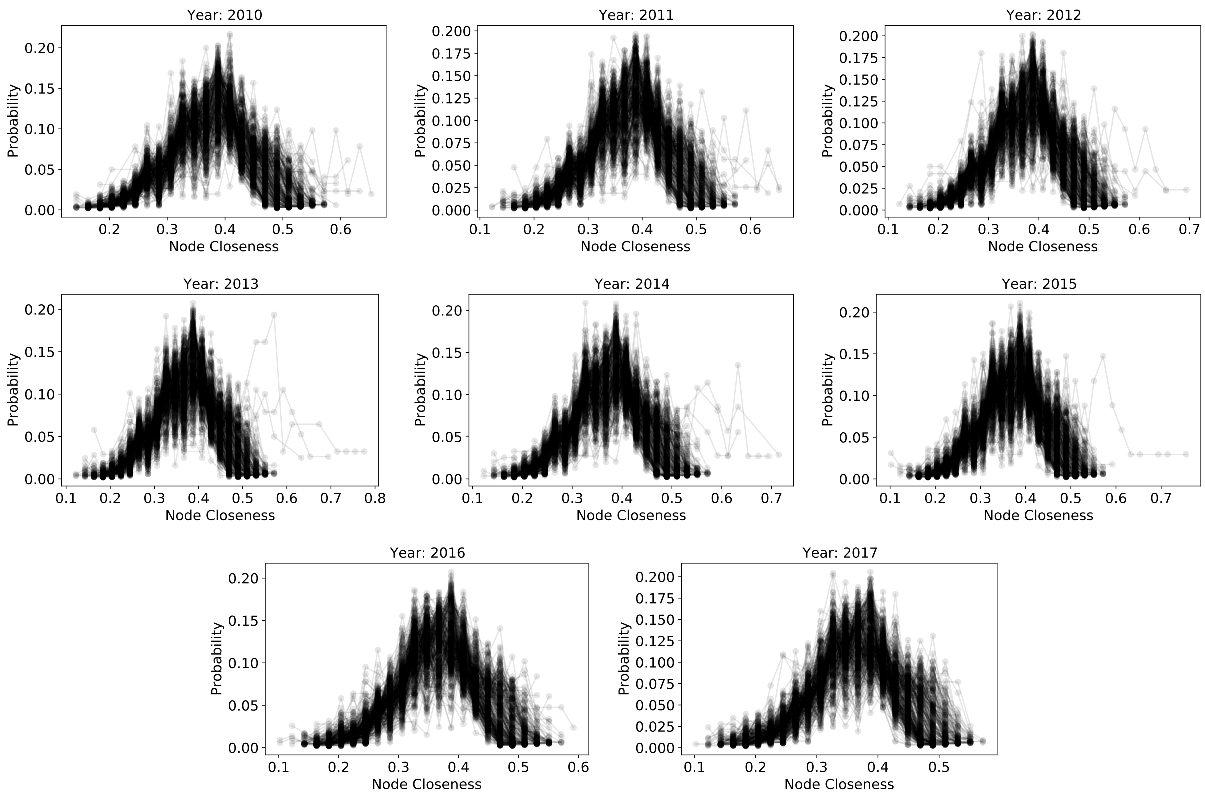

Figure S15: The distribution of node closeness score for each city's job network projection's giant component calculated in a few example years. For each year, one line is plotted for each city.

Supplementary Note 7: Simulating Labor Flows within City

For a given city c, we simulate the employment of occupation E_i and the flow of workers between unemployment U_i and another occupation j using eq. (2) from the main text:

$$\begin{aligned} \dot{E}_j &= -\lambda E_j + \alpha \sum_{i \in Jobs} w_{ij} E_j^{\gamma} U_i^{1-\gamma} \\ \dot{U}_j &= \lambda E_j - \alpha \sum_{i \in Jobs} w_{ij} E_i^{\gamma} U_j^{1-\gamma}. \end{aligned} \tag{1}$$

Here, λ captures the exogenous rate of job match dissolution, w_{ij} measures skill matching between occupations based on occupations' required O*NET skills, and α captures exogenous forces aside from skill matching that shape inter-occupational career mobility.

The simulation starts using the empirical employment distribution on c according to Occupational Employment Statistics (OES) from the US Bureau of Labor Statistics (BLS). We integrate the system using 10,000 iterations of Forward Euler integration with a time step of $\Delta t = 0.5$ to allow the system to reach a steady state. The system is evaluated once it reaches a steady state at the end of the simulation to avoid transient dynamics.

For simulations of employment shocks from automation, the system is integrated for an additional 1,000 iterations after the removal of occupations with exposure to automation above some threshold θ. An occupation's exposure to automation is determined using estimates from [2]. If occupation i has exposure to automation above θ, then we simulate that systemic shock by setting $w_{ji} = 0$ for each $j \neq i$ before the additional simulation and immediately transitioning all workers of i to unemployment. Effectively, if an occupation is automated according to this methodology, then employed and unemployed workers of other occupations cannot transition to employment or unemployment in occupation i. Current employed and unemployed workers of i can transition to other employment opportunities if there skills match the skills required by the new occupation (i.e., w_{ij} is large). In this way, we see how large skill matching complexity (i.e., large w_{eff}) can indicate economic resilience in a labor market since we would expect displaced workers will have an easier time finding new employment opportunities on average.

Supplementary Note 8: Job Network Embeddedness and Worker Wages

Variable	Description
$wage^{c}_{year,i}$	The average annual wage of workers of occupation i in city c in $year$ according to OES data.
$wage^{national}_{year,i}$	The nationwide average wage of occupation i in $year$ according to OES data.
$employment^{national}_{year,i}$	The national employment share of occupation i in $year$.
$employment^{c}_{year,i}$	The employment share (%) of occupation i in city c in $year$ according to OES data.
$w^{c}_{year,i}$	The embeddedness of occupation i in city c in $year$ using OES and O*NET data. See main text for definition.
$bachelors_{year,i}$	The percentage of workers of occupation i with a bachelor's degree nationwide in $year$ according to O*NET data.
$zwage^{c}_{year,i}$	The z-score of $wage^{c}_{year,i}$ compared to the average annual wage of occupation i across all cities in $year$.
$zemployment^{c}_{year,i}$	The z-score of $employment^{c}_{year_i}$ compared to the employment share of occupation i in all cities in $year$.
$zw^{c}_{year,i}$	The z-score of $w^{c}_{year,i}$ compared to the embeddedness of occupation i across all cities in $year$.
$\%wage^{c}_{year,i}$	The percentage change in $wage_{year,i}$ compared to nationwide average annual wage of occupation i in $year$. $100 \cdot (wage^{c}_{year,i} - wage^{national}_{year_i})/wage^{national}_{year,i}$
$\%employment^{c}_{year,i}$	The percentage change in $employment^{c}_{year,i}$ compared to the nationwide employment share of occupation i in $year$. $100 \cdot (employment^{c}_{year,i} - employment^{national}_{year,i})/employment^{national}_{year,i}$
$\%w^{c}_{year,i}$	The percentage change in $w^{c}_{year,i}$ compared to the average embeddedness of occupation i across all cities in $year$. $100 \cdot (w^{c}_{year,i} - \langle w^{c'}_{year,i} \rangle_{c' \in Cities})/\langle w^{c'}_{year,i} \rangle_{c' \in Cities}$

Table S1: Definition of regression variables. Data covers each year from 2005 through 2017.

Dependent Variable: $zwage^{c}_{year,i}$					
Variable	Model 1	Model 2	Model 3	Model 4	Model 5
$zemployment^{c}_{year,i}$	0.000			0.016^{***}	0.002
$bachelors_{year,i}$		-0.064^{***}		-0.021^{***}	-0.020^{***}
$zw^{c}_{year,i}$			0.253^{***}	0.256^{***}	0.249^{***}
$zemployment^{c}_{year,i} \cdot bachelors_{year,i}$					0.033^{***}
$zemployment^{c}_{year,i} \cdot zw^{c}_{year,i}$					-0.006^{***}
$bachelors_{year,i} \cdot zw^{c}_{year,i}$					0.024^{***}
City Fixed Effects	Yes	Yes	Yes	Yes	Yes
Year Fixed Effects	Yes	Yes	Yes	Yes	Yes
R^2	0.315	0.315	0.327	0.327	0.328
adj. R^2	0.315	0.315	0.327	0.327	0.327
$p_{val} < 0.1^{*}$, $p_{val} < 0.01^{**}$, $p_{val} < 0.001^{***}$					

Table S2: Ordinary least-squares regression on the z-score of $wage^{c}_{year,i}$ compared to the average annual wage of occupation i across all cities in each year from 2005 through 2017. See Table S1 for variable definitions and data sources.

Dependent Variable: $zwage^{c}_{year,i}$					
Variable	Model 1	Model 2	Model 3	Model 4	Model 5
$zemployment^{c}_{year,i}$	-0.004^{**}			0.011^{***}	-0.003
$bachelors_{year,i}$		-0.091^{***}		-0.024^{***}	-0.023^{***}
$zw^{c}_{year,i}$			0.273^{***}	0.272^{***}	0.274^{***}
$zemployment^{c}_{year,i} \cdot bachelors_{year,i}$					0.038^{***}
$zemployment^{c}_{year,i} \cdot zw^{c}_{year,i}$					-0.005^{**}
$bachelors_{year,i} \cdot zw^{c}_{year,i}$					-0.004
City Fixed Effects	Yes	Yes	Yes	Yes	Yes
Year Fixed Effects	No	No	No	No	No
R^2	0.356	0.357	0.369	0.369	0.369
adj. R^2	0.355	0.356	0.368	0.368	0.369
$p_{val} < 0.1^{*}$, $p_{val} < 0.01^{**}$, $p_{val} < 0.001^{***}$					

Table S3: Similar to Table S2, but restricting to years prior to the Great Recession. Ordinary least-squares regression on the z-score of $wage^{c}_{year,i}$ compared to the average annual wage of occupation i across all cities in each year from 2005 through 2007. See Table S1 for variable definitions and data sources.

Dependent Variable: $zwage^{c}_{year,i}$					
Variable	Model 1	Model 2	Model 3	Model 4	Model 5
$zemployment^{c}_{year,i}$	0.004^{**}			0.019^{***}	0.000
$bachelors_{year,i}$		-0.054^{***}		-0.021^{***}	-0.019^{***}
$zw^{c}_{year,i}$			0.244^{***}	0.247^{***}	0.234^{***}
$zemployment^{c}_{year,i} \cdot bachelors_{year,i}$					0.039^{***}
$zemployment^{c}_{year,i} \cdot zw^{c}_{year,i}$					-0.008^{***}
$bachelors_{year,i} \cdot zw^{c}_{year,i}$					0.042^{***}
City Fixed Effects	Yes	Yes	Yes	Yes	Yes
Year Fixed Effects	No	No	No	No	No
R^2	0.306	0.307	0.318	0.318	0.318
adj. R^2	0.306	0.306	0.317	0.318	0.318
$p_{val} < 0.1^{*}$, $p_{val} < 0.01^{**}$, $p_{val} < 0.001^{***}$					

Table S4: Similar to Table S2, but restricting to years after the Great Recession. Ordinary least-squares regression on the z-score of $wage^{c}_{year,i}$ compared to the average annual wage of occupation i across all cities in each year from 2012 through 2018. See Table S1 for variable definitions and data sources.

Dependent Variable: $\%wage^{c}_{year,i}$					
Variable	Model 1	Model 2	Model 3	Model 4	Model 5
$\%employment^{c}_{year,i}$	0.177^{***}			0.190^{***}	0.196^{***}
$bachelors_{year,i}$		-4.550^{***}		-4.290^{***}	22.550^{***}
$\%w^{c}_{year,i}$			0.047^{***}	0.037^{***}	0.166^{***}
$\%employment^{c}_{year,i} \cdot bachelors_{year,i}$					0.272^{***}
$\%employment^{c}_{year,i} \cdot \%w^{c}_{year,i}$					0.001^{***}
$bachelors_{year,i} \cdot \%w^{c}_{year,i}$					0.021^{***}
City Fixed Effects	Yes	Yes	Yes	Yes	Yes
Year Fixed Effects	Yes	Yes	Yes	Yes	Yes
R^2	0.276	0.286	0.278	0.288	0.288
adj. R^2	0.276	0.286	0.277	0.288	0.288
$p_{val} < 0.1^{*}$, $p_{val} < 0.01^{**}$, $p_{val} < 0.001^{***}$					

Table S5: Ordinary least-squares regression on the percentage change in $w^{c}_{year,i}$ compared to the average embeddedness of occupation i across all cities in each year from 2005 through 2017. See Table S1 for variable definitions and data sources.

Dependent Variable: $\%wage^{c}_{year,i}$					
Variable	Model 1	Model 2	Model 3	Model 4	Model 5
$\%employment^{c}_{year,i}$	0.230^{***}			0.246^{***}	0.188^{***}
$bachelors_{year,i}$		-4.056^{***}		-3.793^{***}	21.727^{***}
$\%w^{c}_{year,i}$			0.041^{***}	0.027^{***}	0.019
$\%employment^{c}_{year,i} \cdot bachelors_{year,i}$					0.259^{***}
$\%employment^{c}_{year,i} \cdot \%w^{c}_{year,i}$					-0.000
$bachelors_{year,i} \cdot \%w^{c}_{year,i}$					0.007^{**}
City Fixed Effects	Yes	Yes	Yes	Yes	Yes
Year Fixed Effects	No	No	No	No	No
R^2	0.319	0.326	0.320	0.327	0.327
adj. R^2	0.318	0.325	0.319	0.326	0.327
$p_{val} < 0.1^{*}$, $p_{val} < 0.01^{**}$, $p_{val} < 0.001^{***}$					

Table S6: Similar to Table S5, but restricting to years before the Great Recession. Ordinary least-squares regression on the percentage change in $w^{c}_{year,i}$ compared to the average embeddedness of occupation i across all cities in each year from 2005 through 2007. See Table S1 for variable definitions and data sources.

Dependent Variable: $\%wage^c_{year,i}$					
Variable	Model 1	Model 2	Model 3	Model 4	Model 5
$\%employment^c_{year,i}$	0.166^{***}			0.180^{***}	0.225^{***}
$bachelors_{year,i}$		-4.830^{***}		-4.582^{***}	23.358^{***}
$\%w^c_{year,i}$			0.051^{***}	0.041^{***}	0.264^{***}
$\%employment^c_{year,i} \cdot bachelors_{year,i}$					0.283^{***}
$\%employment^c_{year,i} \cdot \%w^c_{year,i}$					0.002^{***}
$bachelors_{year,i} \cdot \%w^c_{year,i}$					0.028^{***}
City Fixed Effects	Yes	Yes	Yes	Yes	Yes
Year Fixed Effects	No	No	No	No	No
R^2	0.265	0.277	0.267	0.279	0.280
adj. R^2	0.265	0.276	0.267	0.278	0.279
$p_{val} < 0.1^{*}$, $p_{val} < 0.01^{**}$, $p_{val} < 0.001^{***}$					

Table S7: Similar to Table S5, but restricting to years after the Great Recession. Ordinary least-squares regression on the percentage change in $w^c_{year,i}$ compared to the average embeddedness of occupation i across all cities in each year from 2012 through 2018. See Table S1 for variable definitions and data sources.

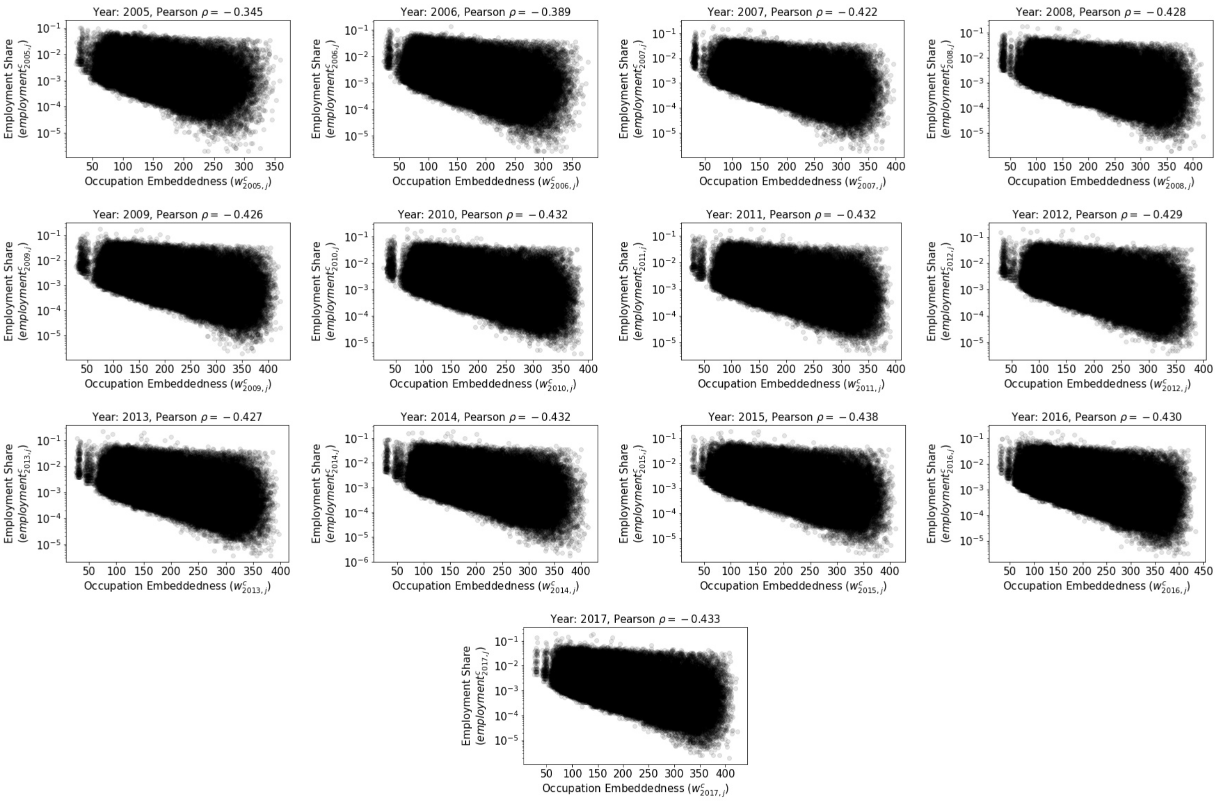

Figure S16: Visualizing each occupation's embeddedness in a city ($w^c_{year,j}$) compared to the occupation's employment share in the city ($employment^c_{year,j}$) in each year from 2005 through 2017.

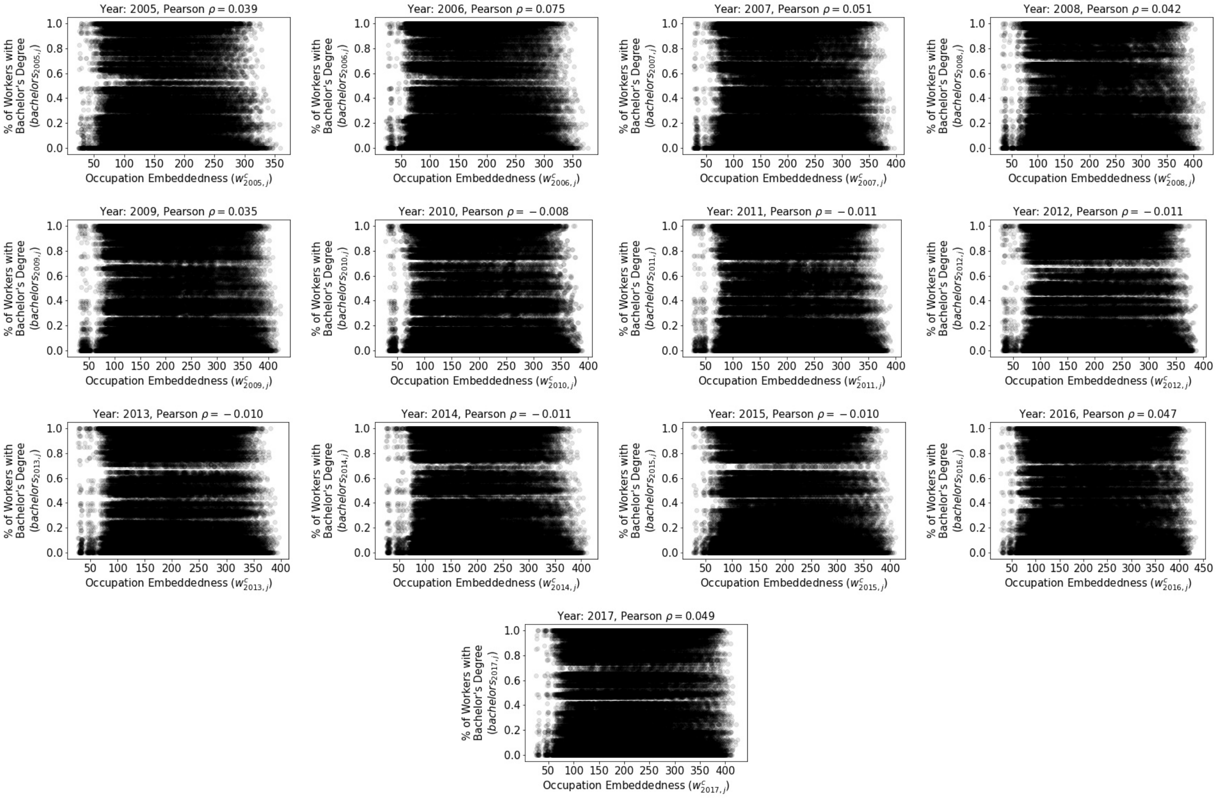

Figure S17: Visualizing each occupation's embeddedness in a city ($w^c_{year,j}$) compared to the national percentage of workers of that occupation with a bachelors degree ($bachelors_{year,j}$) in each year from 2005 through 2017.

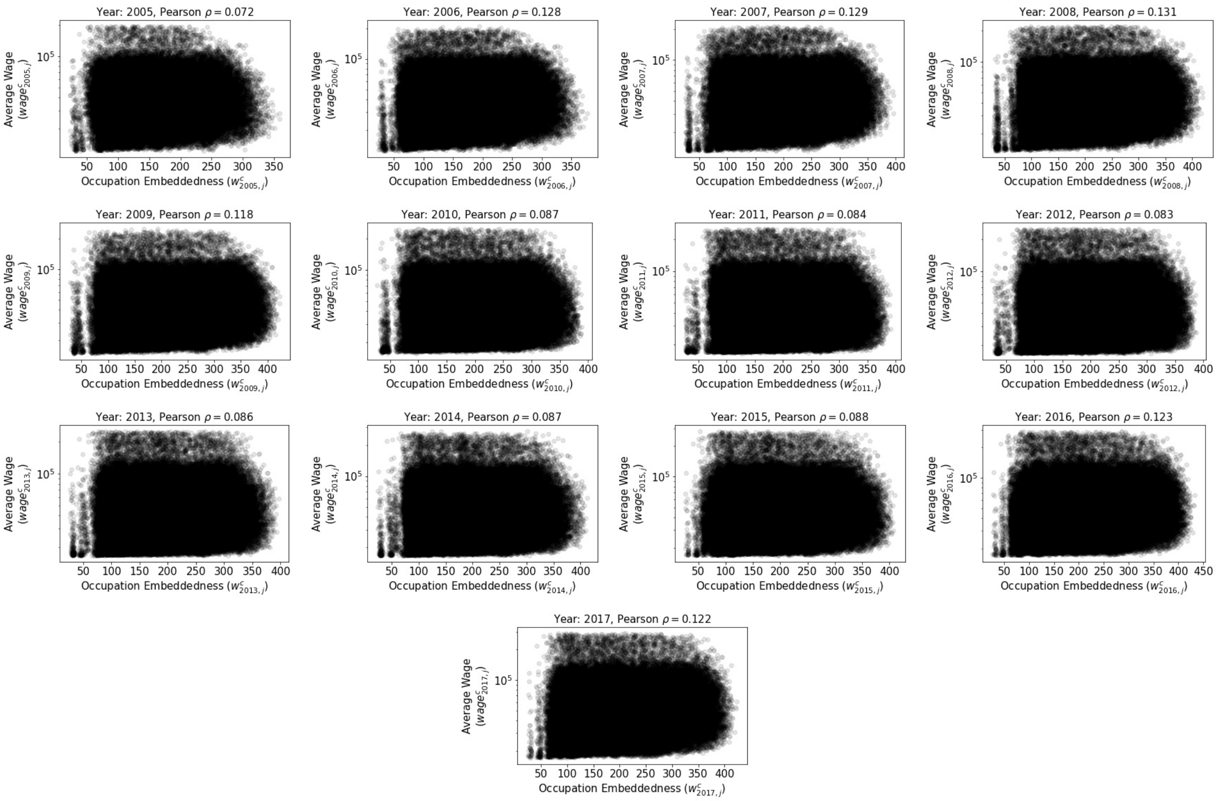

Figure S18: Visualizing each occupation's embeddedness in a city ($w^c_{year,j}$) compared to the occupations average wage in that city ($wage^c_{year,j}$) in each year from 2005 through 2017.

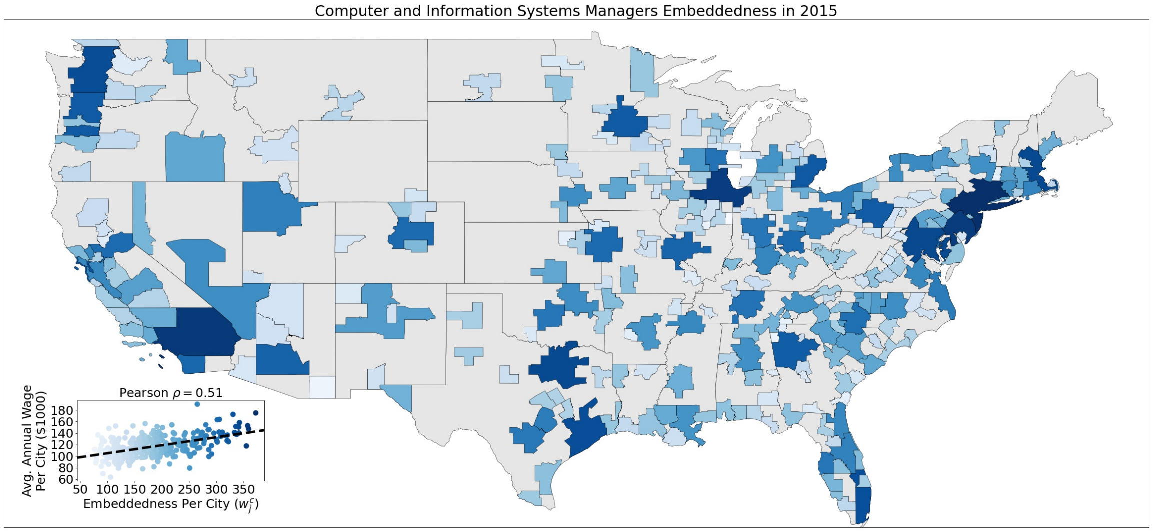

Figure S19: Mapping the embeddedness (w_j^c) of Computer and Information Systems Managers across US cities in 2018. (Inset) Occupation embeddedness compared to the average annual wage ($) across US cities.

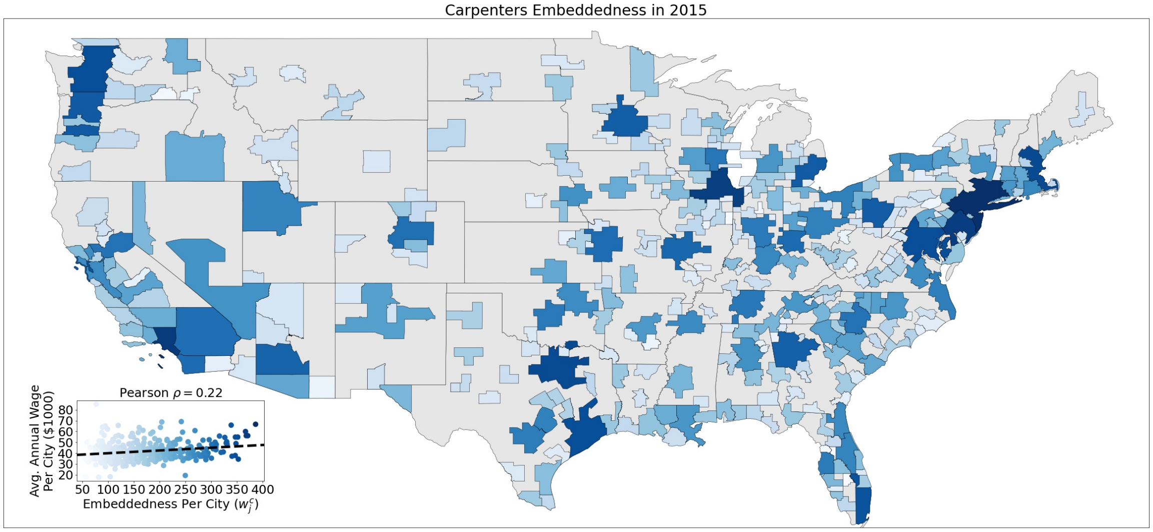

Figure S20: Mapping the embeddedness (w_j^c) of Carpenters across US cities in 2018. (Inset) Occupation embeddedness compared to the average annual wage ($) across US cities.

Supplementary Note 9: Job Connectivity and Wage Bill Growth

Variable	Description
$wagebill^{c}_{year}$	The wage bill (i.e., total wages paid) in city c in *year* according to OES data.
$\%wagebill^{c}_{year1,year2}$	The percentage change of the wage bill in city c in $year2$ compared to that city's wage bill in $year1$.<br>$100 \cdot (wagebill^{c}_{year2} - wagebill^{c}_{year1})/wagebill^{c}_{year1}$
$employment^{c}_{year}$	The total employment in city c in *year* according to OES data.
$\%employment^{c}_{year1,year2}$	The percentage change in city c's total employment in $year2$ compared to in $year1$.<br>$100 \cdot (employment^{c}_{year2} - employment^{c}_{year1})/employment^{c}_{year1}$
$w^{c}_{year,\mathrm{eff}}$	The job connectivity of city c (i.e., w^{c}_{eff}) in *year*. See main text for calculation.
$\%w^{c}_{year1,year2}$	The percentage change in job connectivity w^{c}_{eff} (see main text for definition) in city c in $year2$ compared to in $year1$.<br>$100 \cdot (w^{c}_{year2,\mathrm{eff}} - w^{c}_{year1,\mathrm{eff}})/w^{c}_{year1,\mathrm{eff}}$

Table S8: Definition of regression variables. Data covers each year from 2011 through 2017.

Dependent Variable: $\%wagebill^{c}_{year,2010}$				
Variable	Model 1	Model 2	Model 3	Model 4
$\%employment^{c}_{year,2010}$	1.349^{***} (1.168^{***})		1.234^{***} (1.157^{***})	1.110^{***} (1.107^{***})
$\%w^{c}_{year,2010}$		1.279^{***} (1.637^{***})	0.118^{***} (0.262^{***})	0.116^{***} (0.152^{***})
$\%employment^{c}_{year,2010} \cdot \%w^{c}_{year,2010}$			0.005^{***} (-0.002^{***})	0.003^{***} (-0.001^{***})
Intercept	4.334^{***} (5.367^{***})	8.618^{***} (9.524^{***})	4.370^{***} (4.773^{***})	4.550 (4.677)
Year Fixed Effects	No	No	No	Yes
City Fixed Effects	No	No	No	Yes
R^2	0.869 (0.947)	0.403 (0.264)	0.874 (0.953)	0.971 (0.989)
adj. R^2	0.869 (0.947)	0.403 (0.263)	0.874 (0.953)	0.964 (0.987)
$p_{val} < 0.1^{*}$, $p_{val} < 0.01^{**}$, $p_{val} < 0.001^{***}$				

Table S9: Using employment, wage, and skills data for each year from 2011 through 2017, increasing job connectivity is associated with increasing wage bill. We use 2010 statistics a baseline since 2010 is the first official year after the US Great Recession. See Table S8 for variable definitions. Regression coefficients in black font represent the regression results when outliers (i.e., data points with values beyond four standard deviations of the average value for at least one variable) are removed. Purple font in parentheses represent the regression coefficients when no data points are removed.

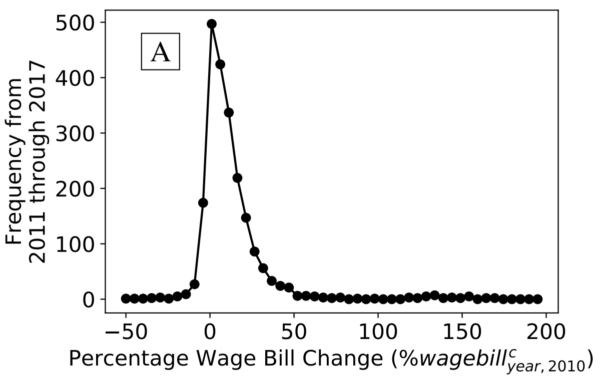

Figure S21: A) Distribution of $\%wagebill^{c}_{year,2010}$ across all cities in each year from 2011 through 2017. B) Same Figure 3E in the main text but including all data points and outliers.

Dependent Variable: $\%wagebill^{c}_{year,year-1}$

Variable	Model 1	Model 2	Model 3	Model 4
$\%employment^{c}_{year,year-1}$	1.260*** (1.118***)		1.008*** (1.018***)	1.086*** (1.039***)
$\%w^{c}_{year,year-1}$		0.688*** (0.834***)	0.224*** (0.266***)	0.070*** (0.106***)
$\%employment^{c}_{year,year-1} \cdot \%w^{c}_{year,year-1}$			0.014*** (0.001*)	0.001 (0.000)
Year Fixed Effects	No	No	No	Yes
City Fixed Effects	No	No	No	Yes
R^2	0.832 (0.930)	0.345 (0.225)	0.837 (0.933)	0.867 (0.945)
adj. R^2	0.832 (0.930)	0.345 (0.225)	0.837 (0.933)	0.852 (0.939)

$p_{val} < 0.1^{*}$, $p_{val} < 0.01^{**}$, $p_{val} < 0.001^{***}$

Table S10: Using employment, wage, and skills data for each year from 2005 through 2017, increasing job connectivity is associated with increasing wage bill. In this regression, we examine year-to-year changes in each variable. See Table S8 for variable definitions. Regression coefficients in black font represent the regression results when outliers (i.e., data points with values beyond four standard deviations of the average value for at least one variable) are removed. Purple font in parentheses represent the regression coefficients when no data points are removed.

Supplementary References

1. Morgan R Frank, Lijun Sun, Manuel Cebrian, Hyejin Youn, and Iyad Rahwan. Small cities face greater impact from automation. *Journal of The Royal Society Interface*, 15(139):20170946, 2018.

2. Carl Benedikt Frey and Michael A Osborne. The future of employment: how susceptible are jobs to computerisation? *Technological forecasting and social change*, 114:254–280, 2017.
